# The CEP5 Peptide Promotes Abiotic Stress Tolerance, As Revealed by Quantitative Proteomics, and Attenuates the AUX/IAA Equilibrium in *Arabidopsis*

**DOI:** 10.1074/mcp.RA119.001826

**Published:** 2020-11-23

**Authors:** Stephanie Smith, Shanshuo Zhu, Lisa Joos, Ianto Roberts, Natalia Nikonorova, Lam Dai Vu, Elisabeth Stes, Hyunwoo Cho, Antoine Larrieu, Wei Xuan, Benjamin Goodall, Brigitte van de Cotte, Jessic Marie Waite, Adeline Rigal, Sigurd Ramans Harborough, Geert Persiau, Steffen Vanneste, Gwendolyn K. Kirschner, Elien Vandermarliere, Lennart Martens, Yvonne Stahl, Dominique Audenaert, Jirí Friml, Georg Felix, Rüdiger Simon, Malcolm J. Bennett, Anthony Bishopp, Geert De Jaeger, Karin Ljung, Stefan Kepinski, Stephanie Robert, Jennifer Nemhauser, Ildoo Hwang, Kris Gevaert, Tom Beeckman, Ive De Smet

**Affiliations:** 1 Division of Plant and Crop Sciences, School of Biosciences, University of Nottingham, Loughborough, United Kingdom; 2 Department of Plant Biotechnology and Bioinformatics, Ghent University, Belgium; 3 VIB Center for Plant Systems Biology, Ghent, Belgium; 4 VIB-UGent Center for Medical Biotechnology, Ghent, Belgium; 5 Department of Biomolecular Medicine, Ghent University, Ghent, Belgium; 6 Department of Life Sciences, POSTECH Biotech Center, Pohang University of Science and Technology, Pohang, Republic of Korea; 7 Centre for Plant Integrative Biology, University of Nottingham, Loughborough, United Kingdom; 8 Department of Biology, University of Washington, Seattle, Washington, USA; 9 Umeå Plant Science Centre, Department of Forest Genetics and Plant Physiology, Swedish University of Agricultural Sciences, Umeå, Sweden; 10 Centre for Plant Sciences, Faculty of Biological Sciences, University of Leeds, Leeds, United Kingdom; 11 Institute for Developmental Genetics, Heinrich-Heine University, Düsseldorf, Germany; 12 Screening Core, Gent, Belgium; 13 Expertise Centre for Bioassay Development and Screening (C-BIOS), Ghent University, Ghent, Belgium; 14 Mendel Centre for Plant Genomics and Proteomics, Central European Institute of Technology (CEITEC), Masaryk University (MU), Brno, Czech Republic; 15 Institute of Science and Technology Austria (IST Austria), Klosterneuburg, Austria; 16 Zentrum für Molekularbiologie der Pflanzen, Plant Biochemistry, University Tübingen, Tübingen, Germany

**Keywords:** Plant biology, Arabidopsis, stress response, signal transduction, developmental biology, hormones, mass spectrometry, label-free quantification, protein degradation, phosphoproteome

## Abstract

Peptides derived from non-functional precursors play important roles in various developmental processes, but also in (a)biotic stress signaling. Our (phospho)proteome-wide analyses of C-TERMINALLY ENCODED PEPTIDE 5 (CEP5)-mediated changes revealed an impact on abiotic stress-related processes. Drought has a dramatic impact on plant growth, development and reproduction, and the plant hormone auxin plays a role in drought responses. Our genetic, physiological, biochemical, and pharmacological results demonstrated that CEP5-mediated signaling is relevant for osmotic and drought stress tolerance in *Arabidopsis*, and that CEP5 specifically counteracts auxin effects. Specifically, we found that CEP5 signaling stabilizes AUX/IAA transcriptional repressors, suggesting the existence of a novel peptide-dependent control mechanism that tunes auxin signaling. These observations align with the recently described role of AUX/IAAs in stress tolerance and provide a novel role for CEP5 in osmotic and drought stress tolerance.

## Teaser

The proteome and phosphoproteome of *CEP5* overexpressing *Arabidopsis* seedlings have been determined. This revealed that CEP5 impacts abiotic stress-related processes. Subsequent genetic, physiological, biochemical, and pharmacological results demonstrated that CEP5-mediated signaling is relevant for osmotic and drought stress tolerance in *Arabidopsis*. Furthermore, CEP5 specifically counteracts auxin effects by stabilizing AUX/IAA transcriptional repressors.

## Graphical Abstract

**Figure:**
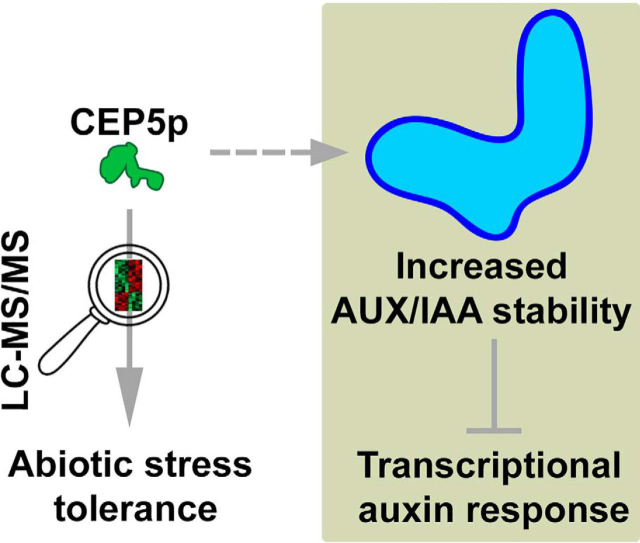

**Highlights**

• Quantitative *Arabidopsis* (phospho)proteomes of C-TERMINALLY ENCODED PEPTIDE 5 (CEP5).
• CEP5 impacts abiotic stress-related processes and counteracts auxin effects.
• CEP5 signaling stabilizes AUX/IAA transcriptional repressors.
• Novel peptide-dependent control mechanism that tunes auxin signaling.

Although peptides derived from non-functional precursors play significant roles in various developmental processes, their involvement in (a)biotic stress signaling is equally important (1, 2, 3). Previously, *Arabidopsis* C-TERMINALLY ENCODED PEPTIDE 5 (CEP5) was shown to play a key role in auxin-mediated primary and lateral root growth and development (4, 5). CEP5 gain-of-function phenotypes with respect to lateral root positioning and patterning are similar to what was observed with altered MONOPTEROS (MP)/ARF5 or BODENLOS (BDL)/IAA12 activity (4, 6), suggesting that auxin-dependent lateral root patterning was disturbed. In addition, CEP family peptides impact the expression of nitrate transporters in the root, signal via XYLEM INTERMIXED WITH PHLOEM 1 (XIP1)/CEP RECEPTOR 1 (CEPR1) and CEPR2, and induce phloem-specific polypeptides in leaves that act as long-distance mobile signals trans-located to the root (7, 8). Recently, it was suggested that CEP-CEPR-dependent signaling controls *Arabidopsis* and *Medicago* root system architecture, gravitropic set-point angle of lateral roots, shoot auxin levels and rootward auxin transport (9). However, based on the diverse expression patterns of *CEP* family peptides (10) and a recently described role in sucrose-dependent enhancement of lateral root growth (11), these peptides likely play important roles beyond nitrogen acquisition.

The phytohormone auxin regulates many plant growth and developmental processes and is prominently involved in lateral root development (12, 13, 14). The core components of the transcriptional auxin response are the AUXIN RESPONSE FACTORs (ARFs), which are transcription factors of which the activity is controlled by AUXIN/INDOLE-3-ACETIC ACID INDUCIBLE (AUX/IAAs) repressor proteins (14). The abundance of these AUX/IAAs is, in an auxin-dependent manner, controlled by AUX/IAA-TRANSPORT INHIBITOR RESPONSE 1 (TIR1)/AUXIN SIGNALING F BOX PROTEIN (AFB) co-receptor complexes that lead to ubiquitin-mediated degradation of these AUX/IAAs (14) (supplemental Fig. S1). To generate auxin-mediated outputs, a complex mechanism involving spatio-temporal expression of *ARFs* and *AUX/IAAs*, variation in auxin sensitivity of TIR1/AFB co-receptor complexes, phosphorylation- and sumoylation-mediated ARF - AUX/IAA interactions, and regulation of proteasome activity is required (15, 16, 17, 18, 19, 20). However, fine-tuning temporal and spatial developmental responses at the protein level most likely requires additional mechanisms to the ones described above. For example, small signaling peptides are important in cell-cell communication to coordinate and integrate cellular functions (21, 22), as seen in the TRACHEARY ELEMENT DIFFERENTIATION INHIBITORY FACTOR (TDIF) - TDIF RECEPTOR (TDR) - BRASSINOSTEROID-INSENSITIVE2 (BIN2) signaling cascade that interferes with ARF - AUX/IAA interactions (18).

Abiotic stresses, such as drought, have a dramatic impact on plant growth, development and reproduction (23), but little is known about the role of auxin in drought responses (24, 25, 26) and even less about the involvement of peptides derived from non-functional precursors (27, 28, 29). AUX/IAAs function as hubs to integrate genetic and environmental information, including drought and osmotic stress (25), and accumulation of auxin in the root system enhances wheat yield under drought (30). Regarding peptides, a prime example is CLAVATA3/EMBRYO-SURROUNDING REGION-RELATED 25 (CLE25), which moves from roots to leaves to transmit a dehydration signal and enhances drought resistance by inducing abscisic acid levels and controlling stomatal closure (29).

Here, we set out to determine CEP5-mediated proteome changes and to explore potential crosstalk between CEP5 and auxin signaling. We demonstrated that CEP5-dependent signaling leads to the stabilization of AUX/IAA transcriptional repressors, arguing for the existence of a novel peptide-dependent control mechanism that contributes to the fine-tuning of auxin signaling. In addition, we assigned a novel role for CEP5 in drought stress response.

## EXPERIMENTAL PROCEDURES

##### Plant materials

The following transgenic lines and mutants were described previously: *pCEP5::nGFP:GUS, CEP5^OE^* and *CEP5^RNAi^* (10), *35S::DII:VENUS* (31), *xip1–1* (32), *cepr2–3* (33), *pDR5::LUC* (34), *IAA19:HA* (18), *pBDL::BDL:GUS* (35), *rpn12a-1* (36), *rpt2a-2* (36), *pRGA::GFP:RGA* (37), *chl1–5* (38), *aux1–2* (39), *aux1–22* (40), *pin2–2/eir1–1* (41), *axr1–30* (42) and *tir1–1 afb1–3 afb2–3* (43) (also see supplemental Table S1).

##### Plant Growth and Treatment Conditions

Plant growth details are described in the Supplementary Information. For proteome and qPCR analyses, seedlings were grown on square Petri plates under continuous light. For phosphoproteome analyses, seedlings were grown in liquid culture. Osmotic stress analyses were performed as described previously (44). Specifically, wild-type and mutant seeds were equally distributed on 14 cm-diameter Petri dishes and seedlings were grown on half-strength Murashige and Skoog (MS with or without indicated concentration of d-mannitol (Sigma-Aldrich)) under a 16-h-day and 8-h-night regime. For short term treatments involving transfer, the growth medium was overlaid with nylon mesh (Prosep, Belgium) of 20 μm pore size to facilitate transfer. The drought-tolerance assay in soil was performed as described previously, with slight modifications (45). Specifically, wild-type and mutant seedlings were randomized in the same tray for 18–21 days before the weight of all pots was equalized. Water was withheld for ∼2 weeks and then plants were re-watered. Plants of each genotype were used to assess survival in three independent experiments. For peptide treatments, media were supplemented with CEP5p^Pro^ (15 amino acid peptide), CEP5p^Hyp^ (15 amino acid, hydroxyprolinated peptide), or mCEP5p^Hyp^ peptide (mutant 15 amino acid, hydroxyprolinated peptide) (supplemental Fig. S2) to concentrations indicated in the text and/or figure legends. For root analysis, seeds were grown vertically on square Petri plates. For the MG132 treatment, seedlings were germinated on ½ MS medium (on square vertical plates), and 4 days after germination (DAG) the seedlings were transferred to ½ MS medium containing 10 μm MG132 (on square vertical plates) for 2 days.

##### Experimental Design and Statistical Rationale for Proteomics

For proteome analyses, the shoots of vertically grown Col-0 and *CEP5^OE^* seedlings (on mesh) at 10 DAG were harvested after removing the root using a scalpel (for each replicate about 1 g of tissue was harvested) and frozen in liquid nitrogen. In total 8 samples were analyzed, with 4 biological repeats for each genotype which is necessary for subsequent statistical analyses. Col-0 was used as the wild-type control. For phosphoproteome analyses, liquid culture-grown (for 5 days after germination) Col-0 wild-type or *CEP5^OE^* seedlings and Col-0 wild-type seedlings treated with 5 μM CEP5p^Hyp^ or 5 μm mCEP5p^Hyp^ for one hour were harvested in three biological replicates (about 1 gram fresh material was harvested for one replicate). Col-0 or Col-0 treated with 5 μm mCEP5p^Hyp^, respectively, were used as controls.

Data filtering and statistical analyses were performed as previously described (46). The original data set with log2-transformed intensities was split into three subsets. The first subset consisted of proteins that were detected in 3 out of 4 biological repeats in both genotypes or phosphopeptides that were detected in 2 out of 3 biological repeats in both genotypes or treatments. This data set with no or few missing values was checked for normal distribution and then submitted for statistical analysis (without applying any imputation), which was performed as described previously (46, 47). A two-sample test with *p* < 0.05 was carried out to test the differences between groups and the centered significant hits were Z-scored and then clustered into groups by a hierarchical clustering analysis based on Pearson correlation, and visualized as heat maps. The second data set, which contained proteins or phosphopeptides only quantified in 2 of the 4 biological replicates or 1 of the 3 biological replicates, respectively, of at least one genotype, was considered as “unreliable” and excluded from further analysis. The proteins that had 0 or 1 value in one genotype and 3 or 4 values in the other genotype or the phosphopeptides that had 0 values in one genotype or treatment and 2 or 3 values in the other genotype or treatment were clustered into the third data set. This data set contained unique hits for one genotype or treatment without any subsequent statistical analysis.

##### Protein Extraction and SCX Fractionation

Protein extraction was performed as previously described (47). The protein pellets were washed with 80% acetone and resuspended in 8 m urea in 50 mm triethylammonium bicarbonate (TEAB) buffer (pH 8). Before the protein concentration was measured using NanoDrop (Thermo Fisher), reduction and alkylation were performed by adding tris(carboxyethyl)phosphine (TCEP, Pierce) and iodoacetamide (Sigma-Aldrich) to final concentrations of 15 mm and 30 mm, respectively, and samples were incubated for 15 min at 30 °C in the dark. For each biological replicate, 1 mg of total protein was pre-digested with EndoLysC (Wako Chemicals, Japan) for 4 h and then digested with trypsin overnight (Promega Trypsin Gold, mass spectrometry grade, Promega) after diluting the samples 8 times with 50 mm TEAB buffer (pH 8). The digest was acidified to pH ≤ 3 with trifluoroacetic acid (TFA) and desalted with SampliQ C18 SPE cartridges (Agilent) according to the manufacturer's guidelines.

SCX fractionation was performed as described (48). Three discs of (1.5 mm diameter) of polystyrene divinylbenzene copolymer with sulfonic acid (Empore™, 3 m) were stacked in a 200 μl pipette tip to make SCX tips. The desalted peptides were fully dried in a vacuum centrifuge and then re-suspended in loading buffer [5% (v/v) acetonitrile, 1% (v/v) TFA]. 100 μg of peptide material in 100 μl loading buffer was loaded on SCX tips which were first rinsed with 100 μl acetonitrile (ACN). Then peptides were eluted by using 20 μl each of the following SCX fractionation buffers: 100 mm ammonium acetate [20% (v/v) acetonitrile, 0.5% (v/v) TFA] (fraction 1); 175 mm ammonium acetate [20% (v/v) acetonitrile, 0.5% (v/v) TFA] (fraction 1); 375 mm ammonium acetate [20% (v/v) acetonitrile, 0.5% (v/v) TFA] (fraction 2). 20 μl elution buffer [80% (v/v) acetonitrile, 5% (v/v) NH_4_OH] (fraction 3) were used twice to elute the remaining peptides. 2 μl 10% formic acid was added to fraction 3 to avoid deamidation. The fractionated peptides were dried under vacuum. Each fraction was dissolved in 30 μl of 2% (v/v) acetonitrile and 0.1% (v/v) TFA immediately prior to LC-MS/MS analysis.

##### Phosphopeptide Enrichment

The proteins were extracted as described above and 500 μg total proteins were trypsin-digested and subjected to vacuum drying before phosphopeptide enrichment as described previously (47) (note that fractionation was not used for these analyses). In brief, the dried eluates were resuspended in 100 μl of loading solvent (80% acetonitrile, 5% TFA) and incubated with 1 mg MagReSyn® Ti-IMAC microspheres (ReSyn Biosciences, South Africa) for 20 min at room temperature. The microspheres were next washed once with wash solvent 1 (80% acetonitrile, 1% TFA, 200 mm NaCl) and two times with wash solvent 2 (80% acetonitrile, 1% TFA). The bound phosphopeptides were eluted with three volumes (80 μl) of a 1% NH_4_OH solution, immediately followed by acidification to pH ≤ 3 with formic acid. Prior to MS analysis, the samples were vacuum-dried and re-dissolved in 50 μl of 2% (v/v) acetonitrile and 0.1% (v/v) TFA.

##### LC-MS/MS Analysis

LC-MS/MS analysis was performed as previously described (47). The sample was loaded on an Ultimate 3000 RSLC nano LC (Thermo Fisher Scientific) where peptides were first separated by a trapping column (made in-house, 100 μm internal diameter (I.D.) × 20 mm, 5 μm beads C18 Reprosil-HD, Dr. Maisch, Ammerbuch-Entringen, Germany) and then loaded on an analytical column (made in-house, 75 μm I.D. × 150 mm, 3 μm beads C18 Reprosil-HD, Dr. Maisch) at a flow rate of 300 nL/min using the following gradient: solvent A (0.1% TFA in water); a linear gradient from 98% solvent A′ (0.1% formic acid in water) to 55% solvent B′ (0.1% formic acid in water/acetonitrile, 20/80 (v/v)) for 170 min; 99% solvent B′ for 5 min. The LC was in-line connected to a Q Exactive mass spectrometer (Thermo Fisher Scientific). The mass spectrometer was operated in data-dependent, positive ionization mode, automatically switching between MS and MS/MS acquisition. MS/MS spectral data were acquired using the following settings: the source voltage was 3.4 kV and the capillary temperature was 275 °C, MS1 was acquired at resolution of 70,000 (at 200 *m*/*z*) and a mass range *m/z* 400–2000, and the top ten of the most intense ions (resolution 17 500 at 200 *m*/z*)* were isolated for MS2 using predefined selection criteria (AGC target 5 × 104 ions, maximum ion injection time 60 ms, isolation window 2 Da, fixed first mass 140 m/z, spectrum data type: centroid, underfill ratio 2%, intensity threshold 1.7xE4, exclusion of unassigned, 1, 5–8, > 8 charged precursors, peptide match preferred, exclude isotopes on, dynamic exclusion time 20 s). HCD fragmentation was used to produce product ions for analysis. The HCD collision energy was set to 25% normalized collision energy and the polydimethylcyclosiloxane background ion at 445.120025 Da was used for internal calibration (lock mass).

MS/MS spectra were searched against the *A. thaliana* proteome database (TAIR10, 34 509 entries, version November, 2014; http://www.arabidopsis.org/) using the MaxQuant software (version 1.5.4.1). Settings for MaxQuant searches were set as follows (47). Trypsin was selected as enzyme setting. Cleavages between lysine/arginine-proline residues were allowed up to two missed cleavages. Carbamidomethylation of cysteine residues was selected as a fixed modification, and oxidation on methionine residues and acetylation at the N terminus of proteins were selected as a variable modification. For the samples enriched for phosphopeptides, phosphorylation of serine, threonine and tyrosine residues was set as an additional variable modification. The mass tolerance for precursor ions was set to 20 ppm for the first search and to 4.5 ppm for fragment ions for the main search. The minimum peptide length was set to 7 amino acids and the false discovery rate for peptide and protein identifications was set to the 1% default setting. The Max LFQ algorithm allowing label-free quantification and the “Matching Between Runs” feature were enabled.

For the quantitative proteome and phosphoproteome analyses, the “ProteinGroups” and “Phospho(STY)sites” output files, respectively, generated by the MaxQuant search were loaded into Perseus software (version 1.5.6.0). The mass spectrometry proteomics data have been deposited to the ProteomeXchange Consortium via the PRIDE (49) partner repository with data set identifier PXD013382. Annotated spectra can be consulted through MS-Viewer: http://msviewer.ucsf.edu/prospector/cgi-bin/mssearch.cgi?report_title=MS-Viewer&search_key=f0tsjn0ruc&search_name=msviewer. The mass spectrometry phosphoproteomics data have been deposited to the ProteomeXchange Consortium via the PRIDE (49) partner repository with data set identifiers PXD017443 (*CEP5^OE^*) and PXD017444 (CEP5p^Hyp^ treatment). Annotated spectra can be consulted through MS-Viewer: http://msviewer.ucsf.edu/prospector/cgi-bin/mssearch.cgi?report_title=MS-Viewer&search_key=6yourm99sc&search_name=msviewer (*CEP5^OE^*) and http://msviewer.ucsf.edu/prospector/cgi-bin/mssearch.cgi?report_title=MS-Viewer&search_key=p5clcllzhc&search_name=msviewer (CEP5p^Hyp^ treatment).

##### In Silico Data Analyses

Venn diagrams were created with the Venny 2.1 online tool (http://bioinfogp.cnb.csic.es/tools/venny). We performed GO categorization using TAIR (https://www.arabidopsis.org/tools/bulk/go/index.jsp), quantifying the number of genes belonging to a GO category *versus* the total number of genes from the input list. We prioritized GO categories that were present at least for 15% of the candidates and that indicated a process to explore.

##### Histochemical GUS assays

For GUS assays, plants were put overnight in 90% acetone, then transferred to a GUS-solution [1 mm X-Glc, 0.5% (v/v) dimethylformamide (DMF), 0.5% (v/v) Triton X-100, 1 mm EDTA (pH 8), 0.5 mm potassium ferricyanide (K_3_Fe(CN)_6_), 0.5% potassium ferrocyanide (K_4_Fe(CN)_6_), 500 mm phosphate buffer (pH 7)] and incubated at 37 °C for GUS staining, and finally washed in 500 mm phosphate buffer (pH 7). The age of seedlings is indicated in the text and/or figure legends. For microscopic analysis, samples were cleared with 90% lactic acid or as described in (50). Samples were analyzed by differential interference contrast microscopy (Olympus BX53) and a stereomicroscope (Leica MZ16).

##### LUCIFERASE Imaging and Expression Analysis

The LUCIFERASE images were taken by a Lumazone machine carrying a CCD camera (Priceton instrument). The CCD camera with macro lens is controlled by WinView/32 software, and *LUCIFERASE* expression movies were taken automatically every 10 min with 10 min exposure time for ∼ 24 h. Before imaging, plates containing ½ MS were sprayed with 1 mmd-Luciferin (Duchefa Biochemie, The Netherlands). The series of pictures were saved in the TIFF format, and subsequently, the expression level of *pDR5::LUC* in 3-day-old seedlings was measured by selecting the region of interest and quantifying the analog-digital units (ADU) per pixel using ImageJ.

##### qPCR Analyses

Details on the experimental set-ups are described in the text or figure legends and primers can be found in Supplementary Information.

##### DII :VENUS Fluorescence Quantification

**Fig. 4:**
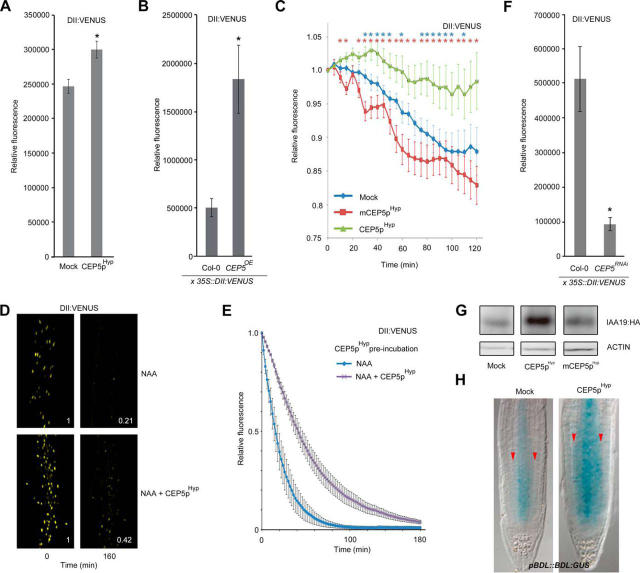
**CEP5 impacts AUX/IAA levels.***A–B*, Relative DII:VENUS protein fluorescence in *35S::DII:VENUS* reporter line following 18 h incubation with 5 μm CEP5p^Hyp^ compared with mock treatment at 5–6 days after germination (n ≥ 83) (*A*) and Col-0 and a *CEP5^OE^* line at 5–6 days after germination (n ≥ 15) (*B*). *C*, DII:VENUS levels upon treatment with CEP5p^Hyp^, mCEP5p^Hyp^ or mock for 120 min (*n* ≥ 4). Graph shows average ± standard error. *, *p* < 0.05 according to Student's *t* test compared with mock (blue) or mCEP5p^Hyp^ (red). With respect to mock *versus* mCEP5p^Hyp^ there was - apart from 15, 30 and 55 min (*p* < 0.05) - no global significant difference. *Note*: no auxin was used in this experiment. *D*, Confocal image of DII:VENUS labeled nuclei from the *35S::DII:VENUS* reporter line in a section of the root that was used for measuring the DII:VENUS protein fluorescence in seedlings treated for 160 min (with 1 μm NAA or with 1 μm NAA and 5 μm CEP5p^Hyp^). Normalized ratio of average top 500 pixel intensity, compared with 0 min, is indicated. *E*, DII:VENUS fluorescence level over time (0–180 min) after transfer to 1 μm NAA, with pre-incubation (18 h) and co-incubation with 5 μm CEP5p^Hyp^ (n ≥ 4). *F*, Relative DII:VENUS protein fluorescence in *35S::DII:VENUS* reporter line in Col-0 and a *CEP5^RNAi^* line at 5–6 days after germination (*n* ≥ 15). *G*, Representative Western blotting of IAA19:HA levels (anti-HA) in 10-day old seedlings grown in the presence of 5 μm CEP5p^Hyp^ or 5 μm mCEP5p^Hyp^ during the whole growth period. Loading control is ACTIN. *Note*: no auxin was used in this experiment. *H*, BDL:GUS protein in representative 6-day old *pBDL::BDL:GUS* root tips after transfer of 4 day old seedlings to mock or 1 μm CEP5p^Hyp^ for 2 days. Red arrowhead marks cortex. In *A*, *B* and *F*, graphs show average ± standard error of indicated sample numbers. *, *p* < 0.05 according to Student's *t* test compared with mock or Col-0. In all cases, mock refers to medium with water as used to dissolve CEP5p^Hyp^.

For DII:VENUS fluorescence measurements in Fig. 4 and in supplemental Figs. S12, S13, and S23, 5–6 day-old seedlings were imaged on a Leica SP5 confocal microscope (Leica, Wetzlar, Germany (514 nm detector: gain value 100, offset value 28.98). Static images of each seedling were taken and fluorescence was quantified by calculating raw integrated density values for each image, measured using FIJI software (51). A zone just above the root hair initiation zone was used for further analyses. Alternatively, seedlings were imaged on an inverted Nikon eclipse Ti-U confocal microscope (Nikon, Japan) with a fixed delay of 2 min over a minimum of 12 h (10× objective, a 515/30 detector using gain value 110, offset value 127). In all cases, background fluorescence was removed using a threshold (which was set manually using the ImageJ “set threshold” tool: threshold was judged to be set when the edges of the nuclei were clearly defined with a minimum of background interference) and only fluorescence coming from the nuclei was quantified. Plots presented in Figs. 4*A*–4*E* and supplemental Figs. S12–S13 show changes in raw integrated density values (how many fluorescent pixels FIJI software counted once the background was subtracted) over time, measured using FIJI software (51). A minimum of 3 seedlings (∼80 nuclei) were independently quantified for each condition. For short term CEP5p treatments, seedlings (*n* = 5–6) were imaged on a Leica SP5 confocal microscope (Leica, Wetzlar, Germany) with a fixed delay of 5 min over a maximum of 8 h (a 514 nm detector using gain value 100%, offset value 28.98, averaged over 4 frames). Fluorescence was quantified as the relative change in raw integrated density values from starting fluorescence over time, measured using FIJI software (51). For Supplemental supplemental Fig. S14, 6 or 7 DAS seedlings were imaged on a Zeiss 710 confocal microscope (514 nm detector: gain value 850, offset 0.00).

##### Yeast Assay

*CEP5p^Pro^* and *mCEP5p^Pro^* were cloned into a pDONR entry vector and then into a modified, single-integration pGAL-Z4 (52) destination vector using Gateway BP and LR technologies. The *Saccharomyces cerevisiae MAT*a W303–1A strain was co-transformed with TIR1 and a β-estradiol-inducible Z4 zinc finger transcription factor (Z_4_EV) (52), whereas the *MAT*α W814–29B strain was co-transformed with YFP-IAA7 or 28 and Z4-inducible CEP5p^Pro^ or mCEP5p^Pro^ following protocols in (53). These two strains were mated, resulting in diploid cells containing all four constructs. Degradation assays were performed using flow cytometry as described in (53). Yeast cultures were treated simultaneously with 1 μm indole-3-acetic acid (in 95% ethanol) and 100 nm β-estradiol (in 95% ethanol). Equivalent volumes of 95% ethanol were used for mock treatments.

##### Auxin Measurements

For auxin measurements, 500 pg 13C6-IAA internal standard was added to each sample (which was generated from 10 day old Arabidopsis seedlings), and extraction and purification was done as previously described (54), with minor modifications. Quantification of free IAA was then performed by gas chromatography - tandem mass spectrometry (GC-MS/MS) as previously described (55).

##### Protein Pull-down and Immunoblotting

The proteins from *IAA19:HA* expressing seedlings were analyzed by 10% SDS-PAGE and visualized with anti-HA (1:2000, Roche) or anti-actin (1:1,000, MP biomedical cat. no. 69100) antibody. Visibility of bands for IAA19:HA in Fig. 4*G* was simultaneously improved using Brightness/Contrast and Level adjustments in Photoshop. To assess AUX/IAA - TIR1 interactions, pull down assays using Streptavidin Biotin:IAA7/17 DII peptides with 3xFLAG:TIR1 were performed in the presence of IAA or IAA + CEP5p^Hyp^, excluding the negative control. IAA and CEP5p^Hyp^ were used at concentrations of 1 μm and 10 μm, respectively. The binding partners were incubated for 1 h at 4 °C, followed by three washes in EB buffer (0.15 m NaCl, 0.5% Nonidet P40, 0.1 m Tris-HCl pH 7.5, 1 mm dithiothreitol, 10 μm MG132) with the IAA (1 μm) and CEP5p^Hyp^ (10 μm) treatments maintained. The 3xFLAG:TIR1 was produced in *N. benthamiana* upon transient expression. Detection was done using anti-FLAG-HRP antibody.

##### Image analyses

**Fig. 2:**
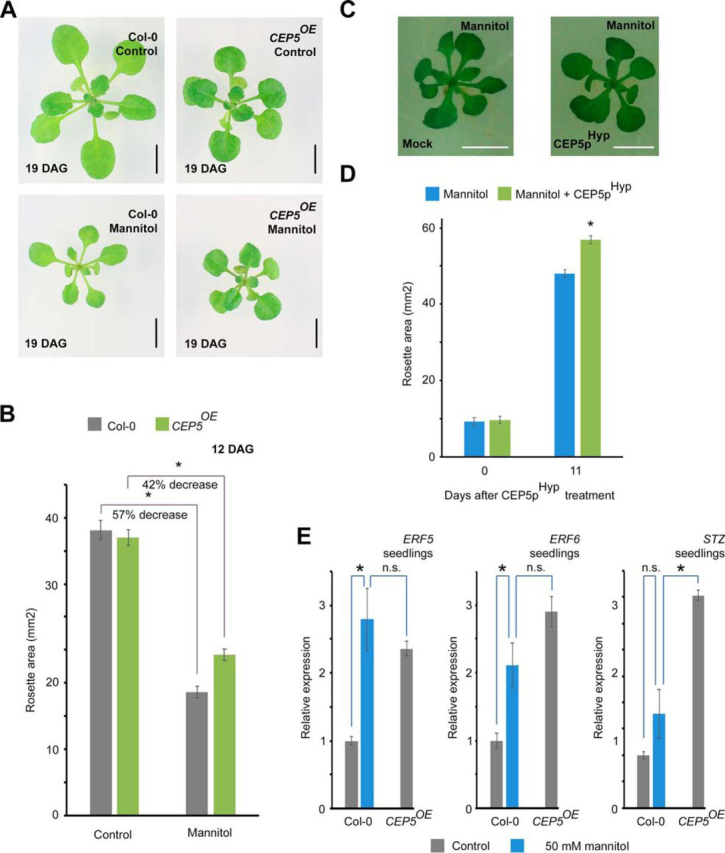
**The CEP5 peptide promotes abiotic stress tolerance by priming seedlings for stress-regulated growth.***A–B*, Col-0 and *CEP5^OE^* plants exposed to osmotic stress (50 mm mannitol). Representative pictures of seedlings at 21 days after stratification (DAS) (*A*) and quantification of rosette size of seedlings at 14 DAS (*B*). Graph shows average of *n* = 23−39 seedlings ± standard error. *, *p* < 0.01 as analyzed by a Student's *t* test. Two-way ANOVA analyses revealed a significant difference (*p* < 0.01) for the Genotype x Treatment interaction. This experiment was repeated 2 times with similar results. Scale bar, 5 mm. *C–D*, Wild-type seedlings at 9 days after sowing (DAS) exposed to osmotic stress (50 mm mannitol) in the absence or presence of synthetic bio-active CEP5p^Hyp^ for 11 days. Representative pictures of seedlings at 20 DAS (*D*) and quantification of rosette size of seedlings at 20 DAS (*E*). Average of n>70 ± standard error. *, *p* < 0.01 as analyzed by a Student's *t* test. This experiment was repeated 2 times with similar results. Scale bar, 5 mm (*E*) *ERF5, ERF6* and *STZ* expression upon osmotic stress and in *CEP5^OE^* plants. Whole seedlings continuously grown on control medium and mannitol (50 mm) until 10 DAS. Average of 3 biological replicates ± standard error. *, *p* < 0.01 as analyzed by a Student's *t* test.

For Fig. 2*B*, 2*D* and supplemental Fig. S6, rosette area was measured in Image J. For Fig. 4*G* and supplemental Fig. S15, we applied an average top 500-pixel intensity measurement starting from a non-saturated image-scan using ImageJ (8-bit image, select ROI, Analyze, Histogram values) and Excel for calculations. Ratios were normalized to the respective loading control (supplemental Fig. S15) and are shown relative to the starting point (0 min).

## RESULTS

##### Proteome and phosphoproteome analyses reveal a potential role for CEP5 in abiotic stress response

**Fig. 1:**
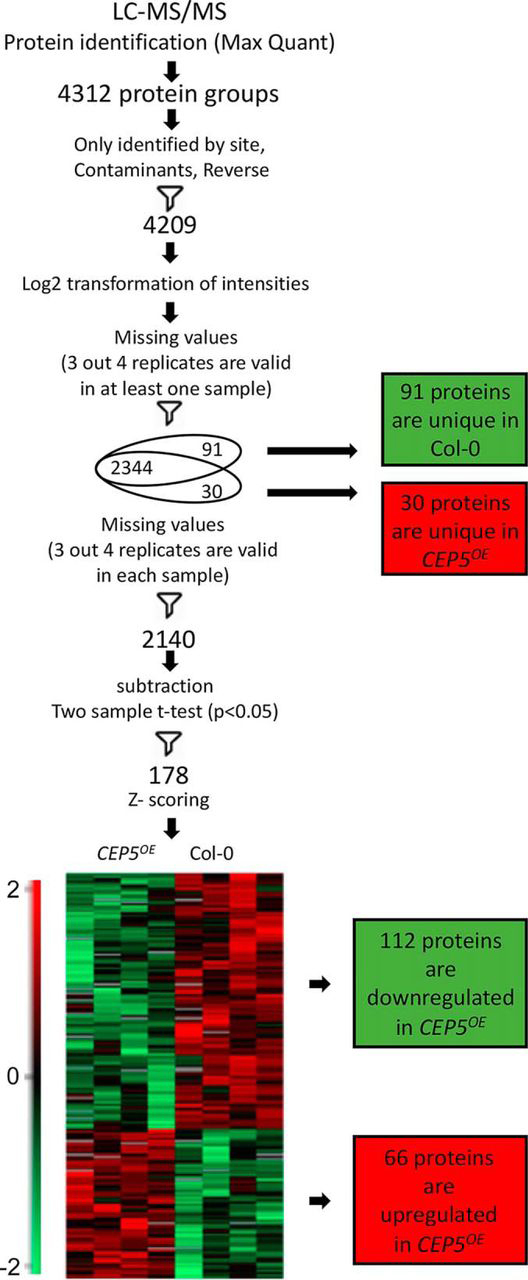
**Workflow of proteome analysis of Col-0 and *CEP5^OE^* shoots following LC-MS/MS.** Venn diagram shows unique proteins (only present in one genotype). Heatmap represents hierarchical clustering of statistically significant proteins (after filtering out the unique ones). Centered Z-scored values of log2-transformed intensity on the heatmap are color-coded according to the color gradient scale. Number of up and downregulated proteins in *CEP5^OE^* is indicated in red and green, respectively.

Although CEP5 has been shown to play a role in shoot and root growth (5, 56, 57), possibly through interaction with the CEPR1/XIP1 and/or CEPR2 receptor kinase (7), very little is known about the downstream molecular effects. To gain insight in the changes downstream of CEP5, we quantified differences in proteomes of wild-type and *CEP5^OE^* shoots using label-free mass spectrometry-based proteomics (Fig. 1). A total of 4209 protein groups were identified and quantified in our analysis (supplemental Table S2). After filtering for proteins that were detected in 3 out of 4 biological repeats in at least one genotype, 2469 proteins were retained for further data analysis. A *t* test (*p* < 0.05) marked 178 proteins with significantly different abundance, including 66 up and 112 down-regulated proteins, in the *CEP5^OE^* line compared with Col-0 (Fig. 1). In addition, we defined unique hits in one genotype as those proteins that had no or only 1 missing value in this genotype, whereas having 3 or 4 missing values in the other genotype. By this criterion, 30 and 91 unique proteins were specifically detected in the *CEP5^OE^* line or in Col-0, respectively (Fig. 1). To gain a global understanding of the data set, we analyzed the gene ontology (GO) annotations in the total set of 299 differential proteins (96 up and 203 down regulated in *CEP5^OE^* in total). This revealed that 30 and 17% of the proteins belonged to the biological processes “response to stress” and “response to abiotic stimulus”, respectively (supplemental Fig. S3).

In addition, we quantified differences in phosphoproteomes of (i) wild-type and *CEP5^OE^* seedlings and (ii) wild-type seedlings treated with CEP5p^Hyp^ or mCEP5p^Hyp^ using label-free mass spectrometry-based proteomics. We identified 386 or 436 phosphorylated peptides that could be mapped on 326 or 354 proteins in *CEP5^OE^* and Col-0 seedlings or in CEP5p^Hyp^ and mCEP5p^Hyp^-treated seedlings, respectively (supplemental Fig. S4 and supplemental Table S3–S4). A similar data analysis as described above for the proteome revealed 18 unique phosphopeptides (present or absent in all three biological replicates of one genotype) and 55 significantly differentially abundant phosphopeptides (*t* test *p* < 0.05) in the *CEP5^OE^ versus* Col-0 data set and 30 unique (present or absent in all three biological replicates of one treatment) and 12 significant phosphopeptides in the CEP5p^Hyp^ *versus* mCEP5p^Hyp^ data set. Also, for these data sets, the biological processes “response to stress” and “response to abiotic stimulus” were well represented (supplemental Fig. S3).

In conclusion, *CEP5^OE^* proteome and phosphoproteome profiling indicated a potential role for CEP5 in abiotic stress responses.

##### CEP5^OE^ and CEP5p^Hyp^-treated Plants Are Osmotic and Drought Stress Tolerant

Because our (phospho)proteome data suggested a connection between CEP5 and abiotic stress, and as drought is a major abiotic stress that reduces crop productivity and yield (58), we investigated a role for CEP5 in drought stress tolerance. When 18-day-old wild-type and *CEP5^OE^* plants were exposed to drought stress for 15 days, we observed that wild-type plants had turned pale and wilted, whereas 20 out of 22 *CEP5^OE^* plants still had some green leaves (supplemental Fig. S5). Furthermore, 21 out of 22 *CEP5^OE^* plants could recover from drought after re-watering (supplemental Fig. S5). Next, because drought and salinity are associated with osmotic stress (44, 59), we tested if overexpression of *CEP5* provided tolerance to osmotic stress. Because mannitol-induced osmotic stress impacts shoot growth and root architecture (44, 60), we exposed *CEP5^OE^* and wild-type seedlings to 50 mm mannitol-containing medium to induce mild osmotic stress, which leads to a reduced rosette size by 50% (61). On mannitol-containing medium, wild-type seedlings displayed stress-induced elongated leaf shapes, whereas *CEP5^OE^* seedlings had normal-looking round leaves (Fig. 2*A*). In addition, compared with control conditions, a less pronounced reduction in rosette area on mannitol-containing medium was observed in *CEP5^OE^* compared with Col-0 (57% in Col-0 and 42% in *CEP5^OE^*; ANOVA *p* value for genotype x treatment <0.01) (Fig. 2*B*). Taken together, our results showed that increased *CEP5* levels resulted in enhanced osmotic and drought stress tolerance. However, this can be a direct effect of CEP5 activity on regulating stress tolerance or an indirect effect through the impact of CEP5 on plant development and consequently reduced soil water usage.

Previously, it was shown that the mature, bio-active CEP5 peptide is likely a 15 amino acid, hydroxyprolinated peptide (referred to as CEP5p^Hyp^) (57). To lower the effect of constitutive high CEP5 levels on overall growth, we exposed already developed ∼7-day-old wild-type seedlings to osmotic stress with or without synthetic bio-active CEP5p^Hyp^. Upon CEP5p^hyp^ treatment, seedlings displayed no osmotic stress-induced elongated leaf shapes (Fig. 2*C*) and showed a significantly larger rosette (Fig. 2*D*). Taken together, our results showed that the synthetic CEP5 peptide is sufficient to protect *Arabidopsis* against osmotic stress.

Finally, we explored if the proposed CEP5 receptor kinases played a prominent role in osmotic stress tolerance. For this, we used the loss-of-function *xip1–1* mutant, which harbors a point mutation that results in the substitution of a serine at position 677 to a phenylalanine in the kinase domain of XIP1/CEPR1 (7, 32) and the loss-of-function *cepr2–3* mutant, which contains a frameshift and subsequent early stop codon around the T-DNA insertion site in *CEPR2* (33). However, the *xip1–1 cepr2–3* double mutant did not display a significant difference with respect to rosette size reduction upon osmotic stress treatment compared with Col-0 (supplemental Fig. S6), which indicates that—at least in this context—CEP5 acts independently of the CEPRs.

##### CEP5^OE^ seedlings are primed for osmotic stress

*CEP5* is expressed throughout the seedling and adult plant (56, 57). But, because high levels of CEP5 protect *Arabidopsis* against osmotic stress, we tested whether *CEP5* expression is regulated by osmotic stress. The *CEP5* expression level did not change significantly upon short-term (up to 24 h) exposure of seedlings to mild osmotic stress (50 mm mannitol), both in roots and shoots (supplemental Fig. S7A–S7B). However, in seedlings subjected to mild osmotic stress (50 mm mannitol) for a longer term (8 days), we observed a small, but significant, increase in the *CEP5* expression level (supplemental Fig. S7C). In contrast, in seedlings exposed to more severe osmotic stress (150 mm mannitol) for a longer term (8 days), we observed a down-regulation in *CEP5* expression levels (supplemental Fig. S7D). Taken together, this suggested that *CEP5* expression is controlled by osmotic stress, but that the duration and intensity of the response affects the outcome.

Next, because we observed that already under control conditions *CEP5^OE^* seedlings displayed smaller, dark green and compact leaves, a hallmark for stressed plants (44) (Fig. 2*A*), we hypothesized that *CEP5^OE^* seedlings are primed for osmotic stress. To control growth under osmotic stress, expression of the transcription factors *ETHYLENE RESPONSE FACTOR 5* (*ERF5*) and *ERF6* is induced very early upon osmotic stress and directly induces the expression of other stress-related transcription factors, such as *SALT TOLERANCE ZINC FINGER* (*STZ*) (62, 63). Indeed, *CEP5^OE^* seedlings showed increased expression of *ERF5*, *ERF6* and *STZ* in control conditions when compared with wild-type. These elevated levels of expression were similar or higher to the level of expression observed in wild-type upon exposure to mannitol stress, and could not be further up-regulated by exposure to mannitol stress (Fig. 2*E* and supplemental Fig. S8). Thus, CEP5 positively affects the expression of stress-regulated genes associated with growth, and thus primes plants for osmotic stress already under unstressed conditions.

##### CEP5 Affects Transcriptional Auxin Response

**Fig. 3:**
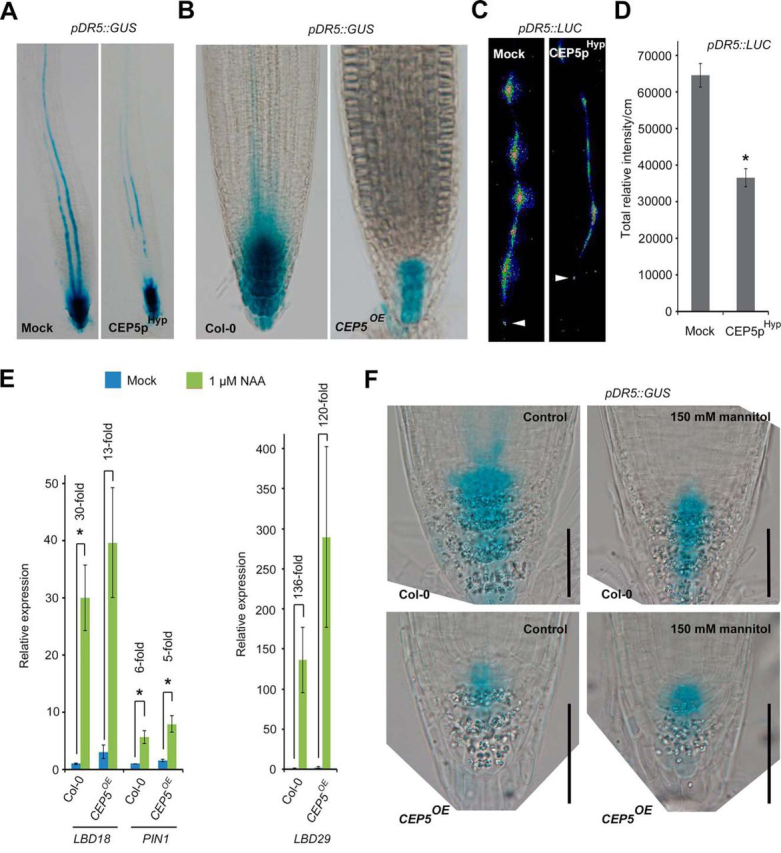
**CEP5 impacts auxin response.***A–B*, Representative pictures for *pDR5::GUS* activity in the primary root tip of 5-day old seedlings transferred to mock or 1 μm CEP5p^Hyp^ for 4 days (*A*) or in the root tip of Col-0 and *CEP5^OE^* at 7 days after germination (*B*). C–D, Representative pictures of *pDR5::LUC* in the root of 3-day old seedlings treated with mock or CEP5p^Hyp^. Arrowhead indicates root tip (*C*). Total relative LUC activity/cm in *pDR5::LUC* following 1 μm CEP5p^Hyp^ treatment (*D*). Graph shows average ± standard error. *, *p* ≤ 0.05 according to Student's *t* test compared with mock. In *A–D*, mock refers to medium with water as used to dissolve CEP5p^Hyp^. *E*, Auxin-inducible expression of *LBD18, LDB29,* and *PIN1* (as determined through qRT-PCR analysis) in 5 day old Col-0 and *CEP5^OE^* seedling roots treated with 1 μm NAA or mock (DMSO) for 6 h (3 biological repeats). Graphs show average ± standard error. *, *p* ≤ 0.05 according to Student's *t* test compared with mock. Fold change of mock *versus* NAA treatment is indicated. Two-way ANOVA analyses revealed a significant difference (*p* < 0.05) for the Genotype x Treatment interaction for *LBD18* fold changes. *F*, Representative pictures for *pDR5::GUS* activity in the primary root top of 8 day old Col-0 or *CEP5^OE^* seedlings grown on control medium or medium containing 150 mm mannitol. This experiment was repeated 2 times with similar results (*n* = 9–18 for each biological replicate). Scale bar, 0.05 mm.

We next sought to identify the CEP5-associated mechanisms mediating drought and osmotic stress tolerance. Although our (phospho)proteome data indicated a CEP5-mediated regulation of abiotic stress-related proteins, such as SNRK2.2 (64, 65), we decided to explore a possible connection between CEP5 and auxin. Specifically, because other loss and gain-of-function CEP5 phenotypes include auxin-mediated control of root architecture (57) and because the phytohormone auxin regulates many plant growth and developmental processes, including osmotic and drought stress tolerance (12, 13, 14, 25, 66, 67). To evaluate to what extent CEP5 affects the transcriptional auxin response, we made use of available auxin-responsive *DR5*-based markers (34, 68) and focused on the root tip as a more tractable system for such analyses (69). We observed reduced activity of the auxin response marker *pDR5::GUS* in the root tip and in the basal meristem following CEP5p^Hyp^ treatment and in the *CEP5^OE^* line (Fig. 3*A*–3*B*). Similarly, the overall average intensity of the *pDR5::LUC* signal, which also marks events associated with lateral root development (34), was severely reduced in the root (Fig. 3*C*–3*D*), supporting our observations with *pDR5::GUS*. We could further confirm the impact on the transcriptional auxin response through analyzing the auxin-inducible expression of root-expressed genes, such as *LOB DOMAIN-CONTAINING PROTEIN 18* (*LBD18*), *LBD29*, and *PIN-FORMED 1* (*PIN1*), which was reduced in auxin-treated *CEP5^OE^* roots compared with the control (Fig. 3*E*). Similarly, we showed reduced auxin-inducibility of *ARF19*, *PIN1* and *LBD29* expression in *xip1–1* compared with the control (supplemental Fig. S9). However, this might also be because of the overall different root architecture of *xip1–1* compared with Col-0 (supplemental Fig. S14B). Finally, wild-type and *CEP5^OE^* seedlings expressing *pDR5::GUS* were exposed to mock and osmotic stress. This revealed that mannitol treatment affects the *pDR5::GUS* expression pattern and intensity in Col-0 root tips, and that this pattern and intensity are similar to the untreated *CEP5^OE^* line (Fig. 3*F*). Furthermore, mannitol treatment of *CEP5^OE^* does not further reduce *pDR5::GUS* expression in the root tip (Fig. 3*F*). Together with the elevated expression levels of *ERF5*, *ERF6* and *STZ*, this further suggests that *CEP5^OE^* seedlings are primed for osmotic stress response and that CEP5 - possibly through XIP1/CEPR1 and/or CEPR2 - affects auxin-responsive gene expression in the root. Because *pDR5::GUS* expression is similarly affected in the shoot of our *CEP5^OE^* line (Supplemental Fig. S10), we assume that similar pathways are at work in the root and in the shoot.

##### CEP5 Leads To Stabilization of AUX/IAAs

Transcriptional responses to auxin depend principally on the auxin-activated SKP1-CUL1/CDC53-F-BOX (SCF)^TIR1/AFB^-dependent proteasome-mediated degradation of AUX/IAAs (14). The activity level of the SCF^TIR1/AFB^ complex and/or auxin concentration can be inferred from the decrease in DII:VENUS fluorescence levels in the root (31, 70). In the presence of CEP5p^Hyp^ and in a *CEP5^OE^* line, DII:VENUS fluorescence was significantly increased compared with the control, and this did not appear to be caused by an equally strong transcriptional up-regulation of *DII:VENUS* expression (Fig. 4*A*–4*B* and supplemental Fig. S11). Moreover, for CEP5p^Hyp^, a stabilization of DII:VENUS was already observed within 35 min, whereas for mock or mCEP5p^Hyp^-treated seedlings a gradual decrease in DII:VENUS signal was observed (Fig. 4*C*). To assess if CEP5 can also interfere with auxin-mediated degradation of DII:VENUS, we co-incubated auxin (IAA or NAA) with CEP5p^Hyp^. This resulted in a significant delay of DII:VENUS degradation compared with auxin alone, whereas mCEP5p^Hyp^ did not affect DII:VENUS degradation (Fig. 4*D*–4*E* and supplemental Fig. S12).

Next, we checked if controlling DII:VENUS levels is a general function for the CEP family. However, the related CEP1p^Hyp^, surprisingly, only had a minor, not significant impact on DII:VENUS fluorescence (supplemental Fig. S13). Furthermore, the *CEP5^RNAi^* line already displayed significantly lower DII:VENUS levels than the control (Fig. 4*F*), which did not appear to be caused by an equally strong transcriptional down-regulation of *DII:VENUS* expression (supplemental Fig. S11). Taken together, it appears that not all CEP family members impact DII:VENUS stability. We also investigated DII:VENUS levels in the loss-of-function *xip1–1* mutant. The *xip1–1* mutant displayed reduced DII:VENUS levels in the root tip (supplemental Fig. S14A), but this might also be associated with the overall altered root architecture (supplemental Fig. S14B).

To validate that CEP5 also affects full length AUX/IAAs, we analyzed plants expressing *35S::IAA19:HA* (18) and *pBODENLOS(BDL)::BDL:GUS* (35). Indeed, CEP5p^Hyp^-treatment of these seedlings resulted in a (quick) stabilization or accumulation of IAA19:HA or BDL:GUS compared with mCEP5p^Hyp^ or mock treatment as revealed by Western blot analysis (increased band intensity) or GUS staining (increased intensity and expanded domain), respectively (Fig. 4*G*–4*H* and supplemental Fig. S15). Interestingly, accumulation of more stable BDL in gain-of-function *bdl* plants results in similar lateral root phenotypes as observed for increased *CEP5* levels (4, 6), further supporting that CEP5 affects AUX/IAA levels and disturbs auxin-dependent growth and development. Furthermore, the CEP5-mediated stabilization of IAA19 is likely an additional layer to control auxin response under abiotic stress conditions, in addition to the DEHYDRATION-RESPONSIVE ELEMENT BINDING PROTEIN 2 (DREB2A) and C-REPEAT/DRE BINDING FACTOR 1 (CBF1)-mediated control of *IAA19* expression under abiotic stress conditions (25).

##### CEP5 Does Not Affect Auxin Levels and Does Not Require Auxin Transport for Its Activity

The above results suggested that CEP5 counteracts auxin activity by (quickly) affecting AUX/IAA levels, either directly through interfering with signaling/degradation components or indirectly through affecting (free) auxin levels and/or auxin distribution patterns. Because auxin response and DII:VENUS levels are intimately correlated with auxin levels, it is possible that increased or decreased CEP5 levels lead to lower or higher auxin levels, respectively, which in turn would result in decreased or increased auxin response. To investigate this, we compared auxin levels in wild-type, *CEP5^OE^* and *CEP5^RNAi^* seedlings, but this revealed no striking differences in free auxin (IAA, indole-3-acetic acid) content (supplemental Fig. S16). We can however not exclude that our analysis missed local and/or more subtle changes in auxin levels. Next, we wanted to exclude that CEP5 affects auxin uptake and/or transport and consequently (local) auxin accumulation. The similar effect of CEP5 on IAA and NAA-induced DII-VENUS degradation (two auxins with different transport properties) already suggested that CEP5 probably has no direct effect on local auxin uptake and/or transport. To further explore this genetically, we tested sensitivity to CEP5 of the *pin-formed 2* (*pin2*) auxin efflux and *auxin 1* (*aux1*) influx carrier mutants. It was previously shown that *CEP5* overexpression or CEP5p^Hyp^ treatment leads to a significantly shorter primary root compared with control conditions (57). Both *aux1* and *pin2* displayed similar sensitivity to CEP5p^Hyp^ application compared with the wild-type in the primary root growth assay (supplemental Fig. S17). Furthermore, because CEP1 was shown to affect *NITRATE TRANSPORTER* (*NRT*) expression levels (7) and because NRT1.1/CHLORINA1 (CHL1) not only transports nitrate but also facilitates uptake of auxin (71), we evaluated this in the context of CEP5. Although *NRT* expression levels were indeed up-regulated in *CEP5^OE^* seedling roots (supplemental Fig. S18A), we did not observe any obvious insensitivity of *chl1–5* (a knockout mutant for *NRT1.1) (*38) to CEP5p^Hyp^ in our primary root growth assay (supplemental Fig. S18B). Taken together, these observations suggest that CEP5 is likely not directly affecting auxin transport and that NRT1.1 is not directly involved in the CEP5-dependent regulation of the auxin response.

##### CEP5 Interferes with Proteasome Activity

Next, we investigated if CEP5 affects AUX/IAA levels through interfering with auxin signaling and/or AUX/IAA degradation components. The increased AUX/IAA levels could be the consequence of transcriptional down-regulation and/or up-regulation of *TIR1/AFBs* and/or *AUX/IAAs,* respectively. Therefore, we checked their expression levels in a *CEP5^OE^* line or in CEP5p^Hyp^-treated seedlings. This revealed a small increase in *TIR1* and *AFB2* to *AFB5* expression levels in *CEP5^OE^* roots and no obvious effect on *IAA12* and *IAA18* expression in CEP5p^Hyp^-treated seedlings compared with the control (supplemental Fig. S19).

To subsequently assess if CEP5 affects the degradation of AUX/IAAs via interference with the activity of the SCF^TIR1/AFB^ complex *in planta*, we analyzed the effect of CEP5p^Hyp^ on the *auxin resistant 1* (*axr1*) and *tir1/afb* loss-of-function mutants. AXR1 encodes a subunit of a heterodimeric RUB-activating enzyme essential for the activation of the TIR1/AFB F-BOX proteins that function as an auxin receptor (15, 72, 73). Both *axr1–30* and *tir1–1 afb1–3 afb2–3* are less sensitive to CEP5p^Hyp^ treatment in a primary root growth assay, suggesting that a functional SCF^TIR1/AFB^ complex is - at least partially - involved in mediating CEP5 activity, or - alternatively - that these mutants are already saturated in their primary root growth-associated response (supplemental Fig. S20). In addition, CEP5p^Hyp^ does not appear to directly affect the interaction between the AUX/IAA domain II peptide and TIR1 in the presence of auxin (supplemental Fig. S21).

**Fig. 5:**
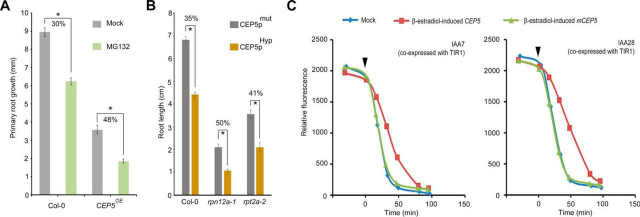
**CEP5 impacts proteasome.***A*, Primary root length inhibition (or decrease) following treatment with MG132 (light green) or mock (gray) for 2 days of 4-day-old Col-0 and *CEP5^OE^* seedlings (*n* = 24–30). Graph depicts average ± standard error. *, *p* < 0.01 as analyzed by a Student's *t* test. The % reduction is indicated. Two-way ANOVA analyses revealed a significant difference (*p* < 0.05) for the Genotype x Treatment interaction. *B*, Primary root length of 11-day-old proteasome subunit mutants *rpn12a-1* and *rpt2a-2 versus* Col-0 (*n* = 12–15). Graph depicts average ± standard error. *, *p* < 0.01 as analyzed by a Student's *t* test. The % reduction is indicated. Two-way ANOVA analyses revealed a significant difference (*p* < 0.05) for the Genotype x Treatment interactions. *C*, Effect of CEP5 peptide on degradation of YFP:IAA7 and YFP:IAA28 in yeast measured as YFP fluorescence. The black arrowhead marks the time point when indole-3-acetic acid (1 μm) and β-estradiol (100 nm) were added. Each data point is an average value of at least 1646 – 3180 events.

Finally, we tested if CEP5 affected degradation of AUX/IAAs by interfering with proteasome activity. Therefore, we first grew seedlings in the presence of the proteasome inhibitor MG132. This showed that *CEP5^OE^* seedlings are more sensitive to MG132 with respect to their primary root growth (Fig. 5*A*). To further strengthen the pharmacological result, we genetically perturbed the proteasome and tested proteasome mutants with respect to their sensitivity to CEP5 treatment. This revealed that *rpt2a-2* (containing a mutation in a subunit of the 19S regulatory particle of the proteasome; gates the axial channel of the 20S core particle and controls substrate entry and product release (36)) and *rpn12a-1* (containing a mutation in a part of the 19S regulatory particle; involved in complex assembly (36)) mutants were more sensitive to CEP5_pHyp_ treatment with respect to primary root growth, compared with the control (Fig. 5*B*). The genetic and pharmacological results support that increased CEP5 levels generate a sensitized condition for loss of proteasome activity. If CEP5 indeed affects a global process, such as the conserved proteasome-mediated protein degradation, we speculated that this should also occur in a heterologous system. Therefore, we used a yeast system engineered to monitor auxin-induced degradation of plant AUX/IAA proteins through fluorescence of YELLOW FLUORESCENT PROTEIN (YFP)-AUX/IAA fusion proteins (53) and we assessed AUX/IAA stability in the presence of CEP5. For this, we integrated the wild-type (*CEP5^Pro^*) and mutant CEP5 15 amino acid mature peptide sequence (*mCEP5^Pro^*) into the yeast genome under a β-estradiol-inducible promoter. It should be noted that CEP5p^Pro^ and CEP5p^Hyp^ give very similar results *in planta*, but differ in their bio-activity (supplemental Fig. S22). We could show that induction of CEP5p^Pro^ was sufficient to negatively affect the auxin-mediated degradation of YFP:IAA7 and YFP:IAA28 in the presence of a functional TIR1 within 100 min, whereas this was unaffected by mCEP5p^Pro^ (Fig. 5*C*). These results indicate that CEP5 interferes with degradation of AUX/IAAs, that this also occurs in the (likely) absence of CEP receptors as shown in yeast, and that this is likely by targeting proteasome activity. It will be interesting to further explore this in detail and identify the precise mode-of-action. Especially because, so far, our results suggested that the effect of CEP5 is limited to auxin response, as we did not observe a similar increase in stability using the RGA:GFP reporter, with REPRESSOR OF GA (RGA) being the counterpart of the AUX/IAAs in gibberellin signaling (37) (supplemental Fig. S23).

## DISCUSSION

Previously, a role for CEPs in regulating aspects of root architecture, namely nitrate-dependent lateral root elongation, was proposed. Specifically, CEPs were suggested to act as root-derived ascending N-demand signals to the shoot, where their perception by CEPRs leads to the production of a putative shoot-derived descending signal that up-regulates nitrate transporter genes in the roots (5, 7, 74, 75). In addition, several CEP peptides were shown to regulate lateral root initiation and primary root growth (4, 5, 76), and enhance lateral root growth in a sucrose-dependent manner (11). Also taking into account the diverse expression patterns of *CEP* family peptides, including expression in aerial organs (10), these peptides likely play important roles beyond nitrogen sensing in the rhizosphere. However, the downstream mechanism was generalized based on selected members from the CEP family and other potential mechanisms have hardly been explored. Here, we expose a novel role for CEP5 in controlling drought and osmotic stress tolerance. In this context, it should be noted that there is crosstalk between nitrogen and drought stress (77), which might explain some of the phenotypes we observed. Furthermore, our genetic, biochemical and pharmacological studies suggest that CEP5 modulates auxin-regulated AUX/IAA stability (supplemental Fig. S1), which - in this way - impacts on auxin-mediated processes, such as drought and osmotic stress tolerance (Fig. 1*B*–1*F*), primary root growth and lateral root initiation (57). The antagonistic relationship between auxin and CEP5 could be important in regulating auxin response thresholds and fine-tuning (sensitive and/or local) auxin responses during growth and development through stabilizing AUX/IAAs. On the one hand, drought-regulated transcription factors will impact on auxin signaling through increasing the expression of *AUX/IAAs* (25), but the expression of *AUX/IAAs* is also positively regulated by auxin (78, 79). Although on the other hand, auxin will lead to the degradation of AUX/IAA proteins (80), which is antagonized by CEP5. Our results suggest that CEP5 impacts the proteasome, but, it remains to be investigated how CEP5 acts directly and possibly specifically on SCF^TIR1/AFB^ and proteasome-mediated AUX/IAA degradation (supplemental Fig. S1). In this context, the identification of CEP receptors, XIP1/CEPR1 and CEPR2 (7) complicates our model. Especially because CEP5 seems to be able to stabilize AUX/IAAs in a heterologous yeast system likely not containing the signaling components identified in *Arabidopsis*. This further supports a direct effect of CEP5 on the SCF^TIR1/AFB^ machinery or downstream degradation processes. In case of a direct interaction with, for example, AUX/IAAs and/or SCF^TIR1/AFB^, CEP5 would be expected to localize in the nucleus, but—so far—this could not be demonstrated. Intriguingly, there are (non-plant) examples of receptors that chaperone their (secreted) ligand into the nucleus (81, 82, 83, 84, 85, 86, 87), and a similar mechanism might exist for the CEP5-XIP1 or CEPR2 pair, reconciling the interaction with a membrane-associated receptor and a direct effect on a nuclear process. Alternatively, CEP5 might act on the cytoplasmic-localized TIR1/AFBs (88). Detailed cell biological assays will be required to convincingly demonstrate one or both above-mentioned possibilities in the future.

Given the expression patterns of the *CEP* family (10) and especially *CEP5*, which appears to mirror areas of increased auxin response (4), the regulation of auxin response may prove to be a general mechanism for some of these small signaling peptides throughout growth and development. However, our data suggest that - at least with respect to stabilizing DII:VENUS - CEP1 is less potent, so there are possibly differences between family members. This is likely because of subtle differences in their mature peptide sequence, as single amino acid changes can impact on bioactivity and/or specificity.

In conclusion, our results support a new mechanism of regulating AUX/IAA stability during growth and development, and future studies are required to expose all the actors involved. In addition, how auxin - CEP5 crosstalk, including the complex gene regulatory networks and AUX/IAA stabilization, impacts abiotic stress tolerance will need to be investigated in more detail.

## DATA AVAILABILITY

The mass spectrometry (phospho)proteomics data have been deposited to the ProteomeXchange Consortium via the PRIDE (49) partner repository with data set identifiers PXD013382, PXD017443 and PXD017444. Annotated spectra can be consulted through MS-Viewer: http://msviewer.ucsf.edu/prospector/cgi-bin/mssearch.cgi?report_title=MS-Viewer&search_key=f0tsjn0ruc&search_name=msviewer, http://msviewer.ucsf.edu/prospector/cgi-bin/mssearch.cgi?report_title=MS-Viewer&search_key=6yourm99sc&search_name=msviewer and http://msviewer.ucsf.edu/prospector/cgi-bin/mssearch.cgi?report_title=MS-Viewer&search_key=p5clcllzhc&search_name=msviewer (CEP5p^Hyp^ treatment).
