# Supplementary material for: The CEP5 Peptide Promotes Abiotic Stress Tolerance, As Revealed by Quantitative Proteomics, and Attenuates the AUX/IAA Equilibrium in Arabidopsis
Source: Mol Cell Proteomics. 2020 Nov 23;19(8):1248–62. doi: 10.1074/mcp.RA119.001826 (PMC8011570; doi:10.1074/mcp.RA119.001826)
Supplement: Supplementary file 1 [file mmc1.zip › 156521_1_supp_527956_qn5nnb.pdf]

SUPPLEMENTARY INFORMATION FOR

The CEP5 peptide promotes abiotic stress tolerance, as revealed by quantitative proteomics, and attenuates the AUX/IAA equilibrium in *Arabidopsis*

Stephanie Smith, Shanshuo Zhu, Lisa Joos, Ianto Roberts, Natalia Nikonorova, Lam Dai Vu, Elisabeth Stes, Hyunwoo Cho, Antoine Larrieu, Wei Xuan, Bert De Rybel, Benjamin Goodall, Brigitte Van De Cotte, Jessica Marie Guseman, Adeline Rigal, Sigurd Ramans Harborough, Geert Persiau, Steffen Vanneste, Gwendolyn K. Kirschner, Elien Vandermarliere, Lennart Martens, Yvonne Stahl, Dominique Audenaert, Jiří Friml, Georg Felix, Rüdiger Simon, Malcolm J. Bennett, Anthony Bishopp, Geert De Jaeger, Karin Ljung, Stefan Kepinski, Stéphanie Robert, Jennifer Nemhauser, Ildoo Hwang, Kris Gevaert, Tom Beeckman, and Ive De Smet

SUPPLEMENTAL FIGURES

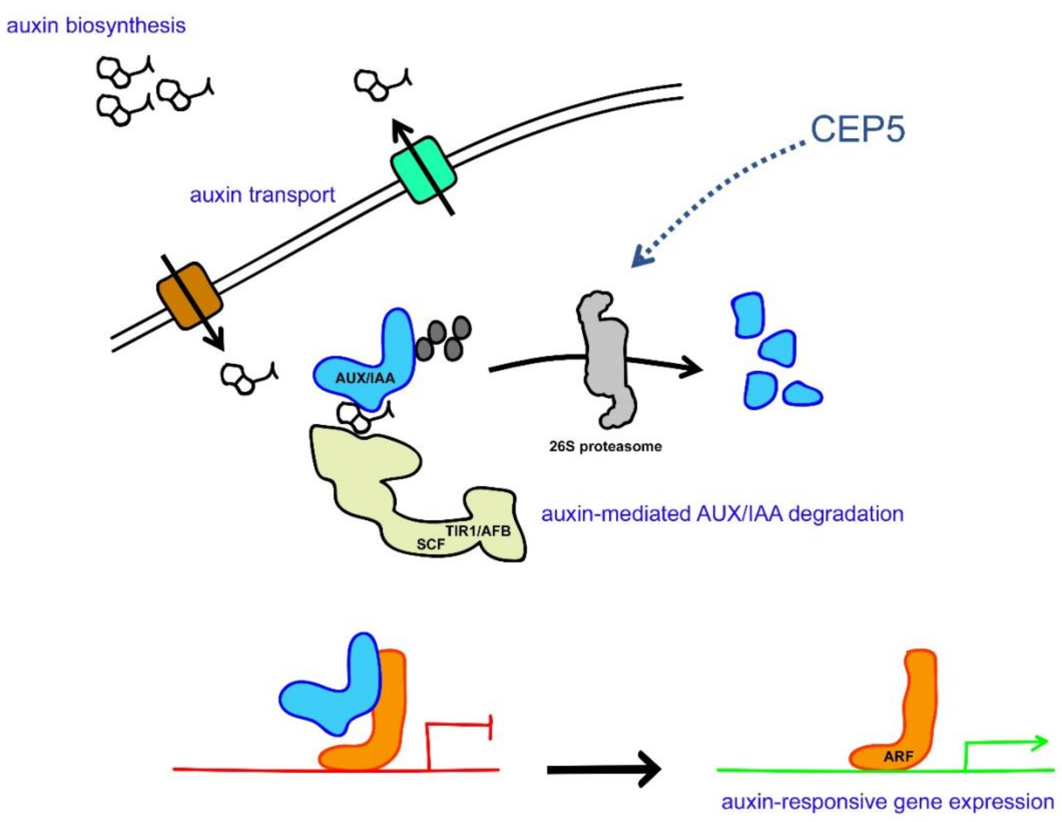

Figure S1. Schematic of basic concepts of auxin response. The key processes checked in our work and the proposed target of CEP5 (dotted line indicates this could be direct or indirect) are indicated. Reproduced/adapted with permission from *Development* [1].

CEP5p^{Pro}	DFRPTTPGHSPGIGH
CEP5p^{Hyp}	DFR{HYP}TT{HYP}GHS{HYP}GIGH
mCEP5p^{Hyp}	DFL{HYP}HT{HYP}GHV{HYP}GISH

Figure S2. Sequences for the synthetic variants of mature 15 amino acid CEP5: unmodified (CEP5p$_{Pro}$), with proline hydroxylation modifications on P4, P7, and P11 (CEP5p$_{Hyp}$), and the hydroxyprolinated mutated CEP5 sequence with four residue substitutions (R3>L, T5>H, S10>V, and G14>S; indicated in red) (mCEP5p$_{Hyp}$).

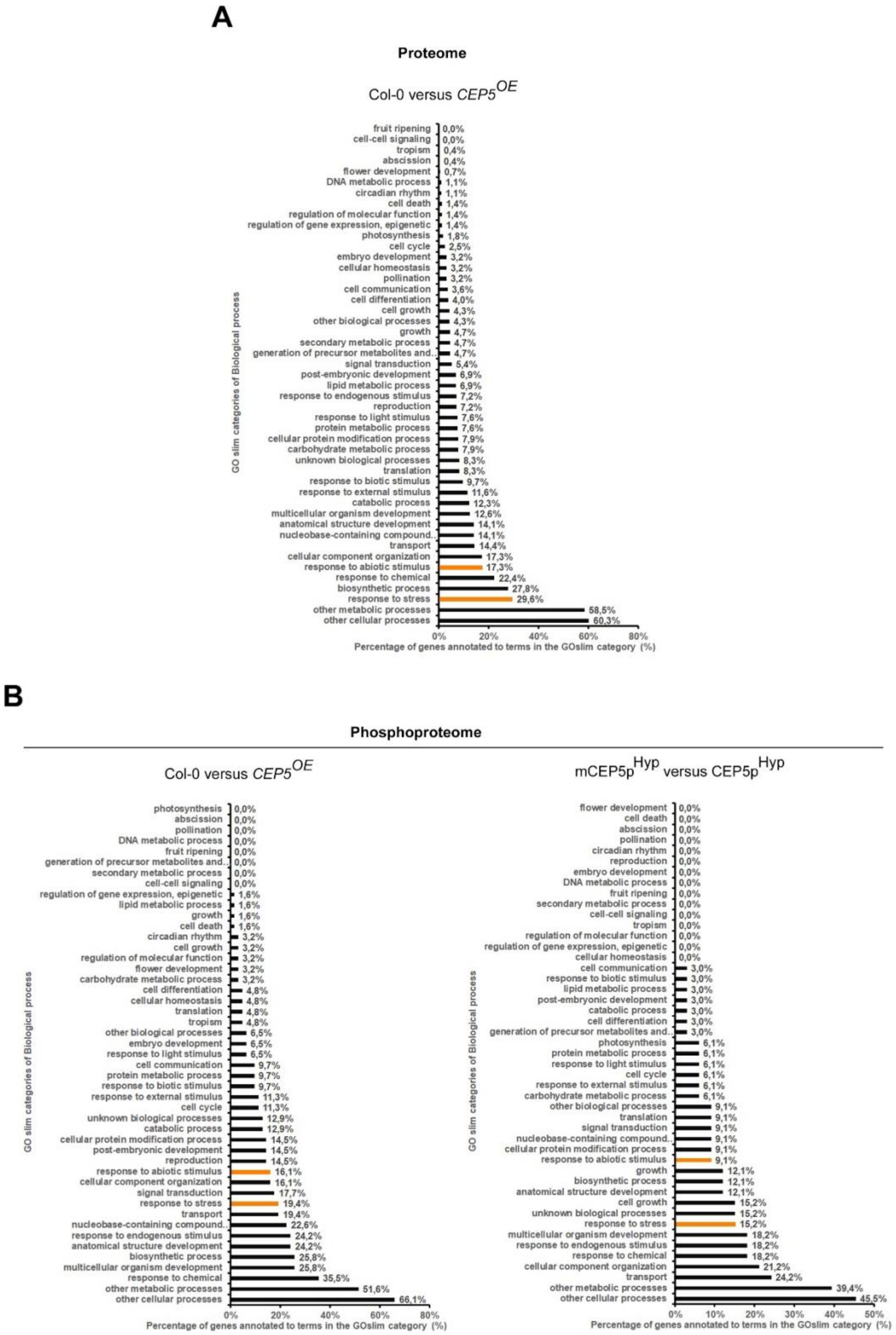

Figure S3. Gene ontology (GO) annotations in **(A)** the total set of 299 differential proteins (96 up and 203 down regulated in *CEP5OE* in total) and **(B)** the total set of differential phosphoproteins in (i) wild type and *CEP5OE* seedlings (29 up and 44 down

regulated in *CEP5*$_{OE}$ in total) and (ii) wild type seedlings treated with CEP5p$_{Hyp}$ and mCEP5p$_{Hyp}$ (8 up and 24 down regulated for CEP5p$_{Hyp}$ in total). We quantified the number of genes belonging to a particular GO category versus the total number of genes from the input list. The percentage of genes annotated to the indicated GO category is indicated. Orange bars highlight the categories "response to stress" and "response to abiotic stimulus".

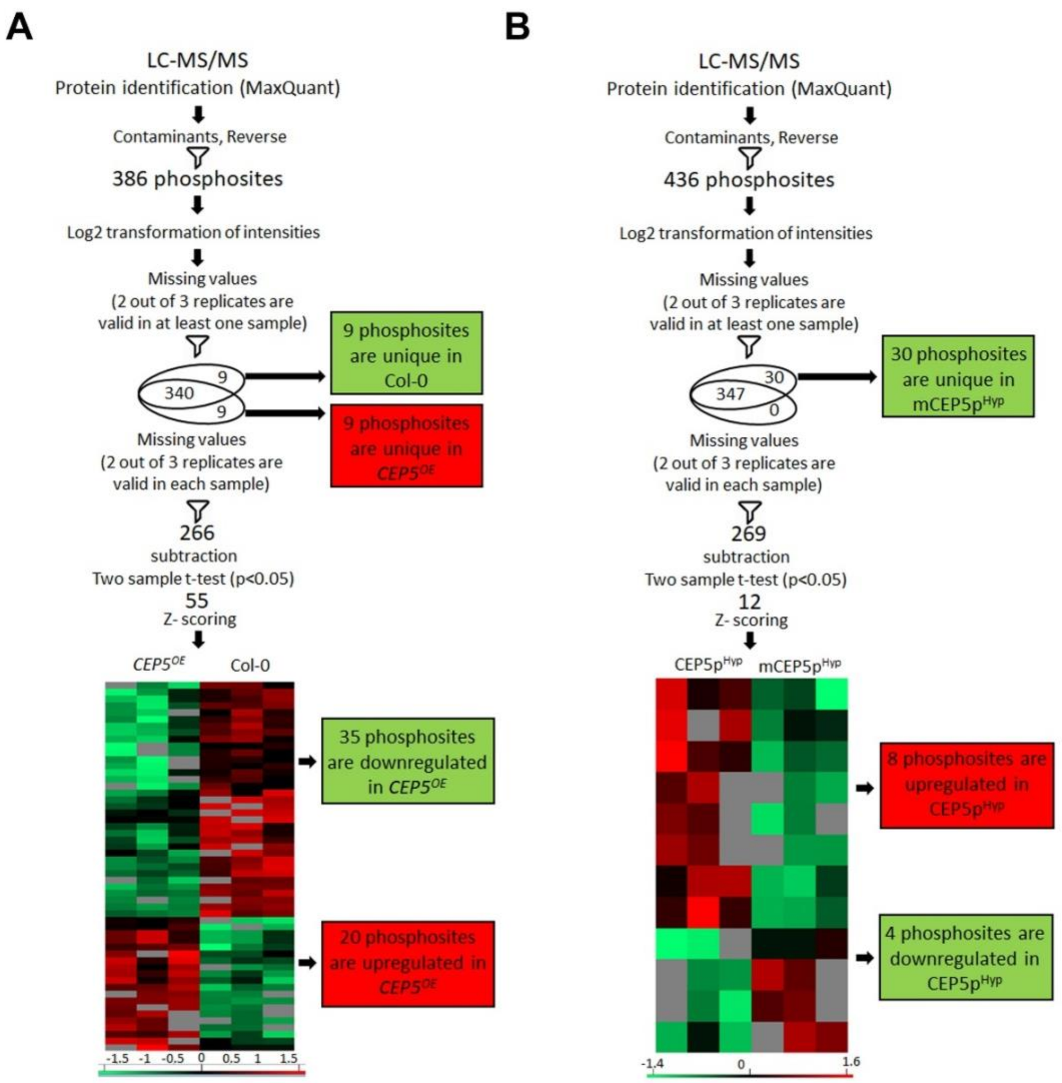

Figure S4. Workflow of phosphoproteome analysis of **(A)** Col-0 and *CEP5OE* seedlings and **(B)** CEP5pHyp and mCEP5pHyp-treated seedlings following LC-MS/MS. Venn diagram shows unique proteins (only present in one genotype). Heatmap represents hierarchical clustering of statistically significant proteins (after filtering out the unique ones). Centered Z-scored values of log2-transformed intensity on the heatmap are color-coded according to the color gradient scale. Number of up and downregulated proteins in *CEP5OE* is indicated in red and green, respectively.

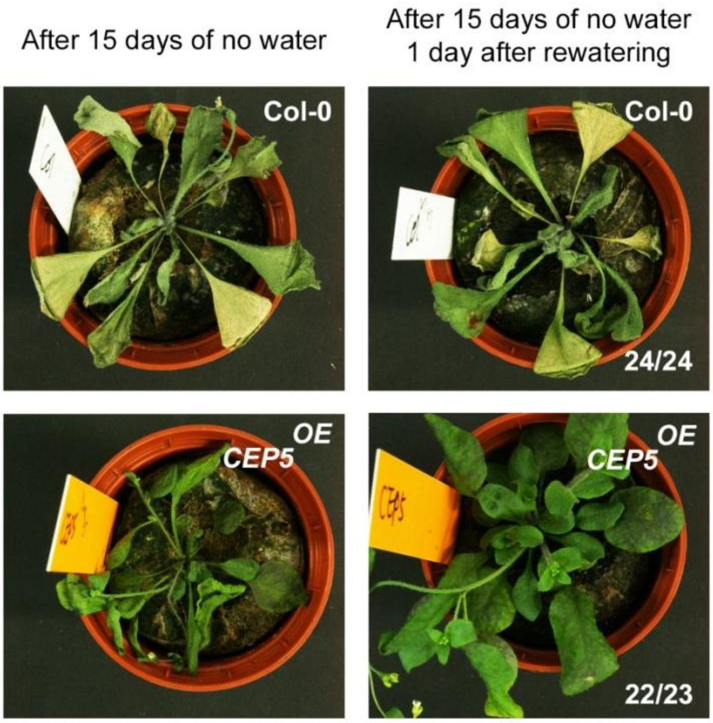

Figure S5. Representative pictures of Col-0 and *CEP5OE* plants subjected to a drought assay in which 18-day-old plants were deprived of water for 15 days (left) and then re-watered for 1 day (right) (n=23-24 plants per line). The number of plants after re-watering that look as shown is indicated. This experiment was repeated 3 times with similar results.

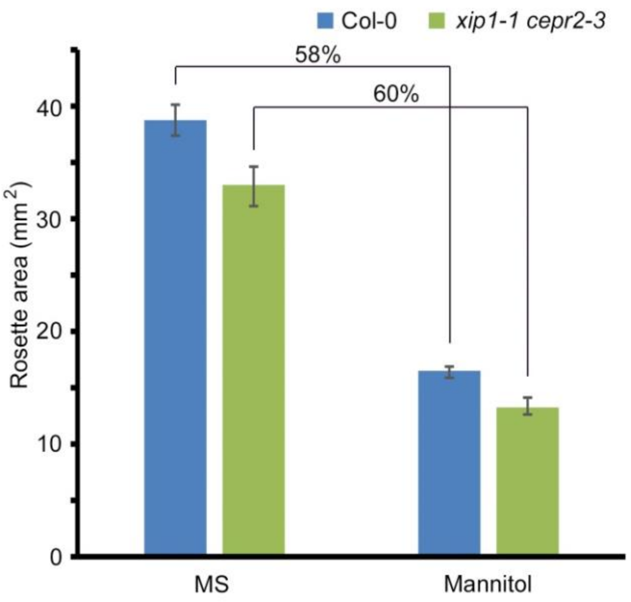

Figure S6. Rosette size of *xip1-1 cepr2-3* upon osmotic stress treatment. Quantification of rosette size of seedlings (n > 18) at 14 DAS. A 2-way ANOVA followed by Tukey's post hoc test revealed no significant difference for genotype x treatment. This experiment was repeated 2 times with similar results.

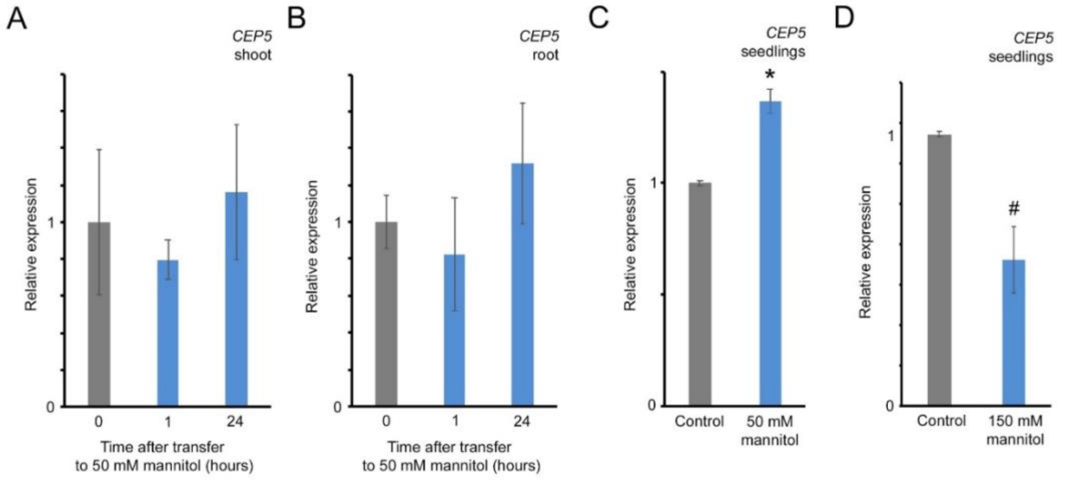

Figure S7. *CEP5* expression upon osmotic stress (50 mM mannitol for indicated time). **(A-B)** Seedlings at 10 DAS transferred from control medium to mannitol (50 mM). *CEP5* expression in shoot (A) and root (B). *, Student's t-test did not reveal significant differences with p-value < 0.05. **(C)** Whole seedlings continuously grown on control medium and mannitol (50 mM) at 10 days after stratification (DAS). *, Student's t-test with p-value < 0.05. **(D)** Whole seedlings continuously grown on control medium and mannitol (150 mM) at 10 DAS. #, Student's t-test with p-value = 0.06. All graphs show average of 3-4 biological replicates ± standard error.

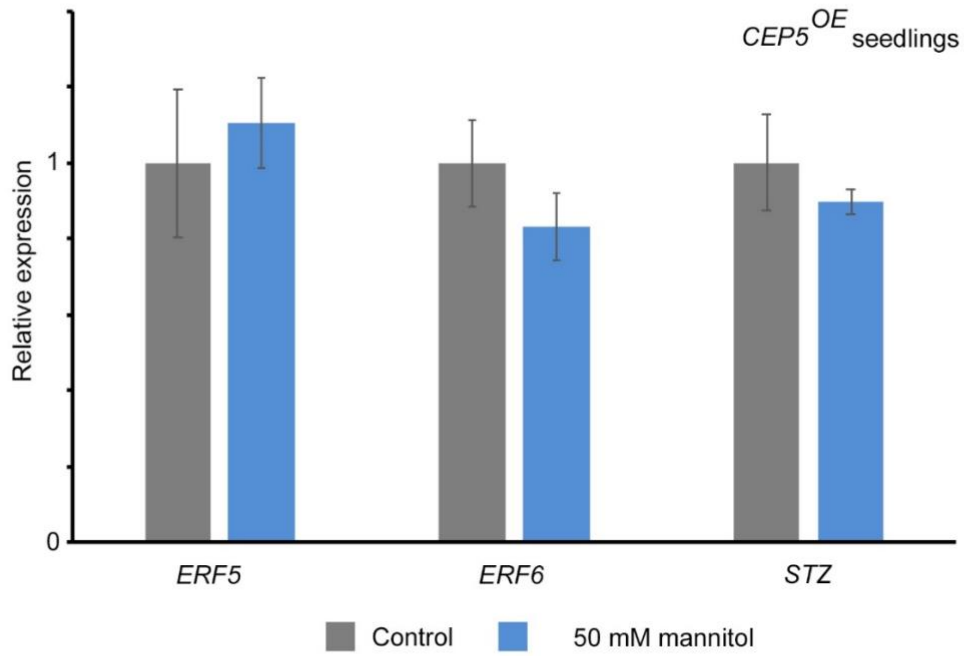

Figure S8. *ERF5, ERF6* and *STZ* expression upon osmotic stress (whole seedlings continuously grown on control medium and mannitol (50 mM) until 10 days after stratification (DAS). Average of 3 biological replicates ± standard error. No significant differences based on Student's t-test with p-value <0.05.

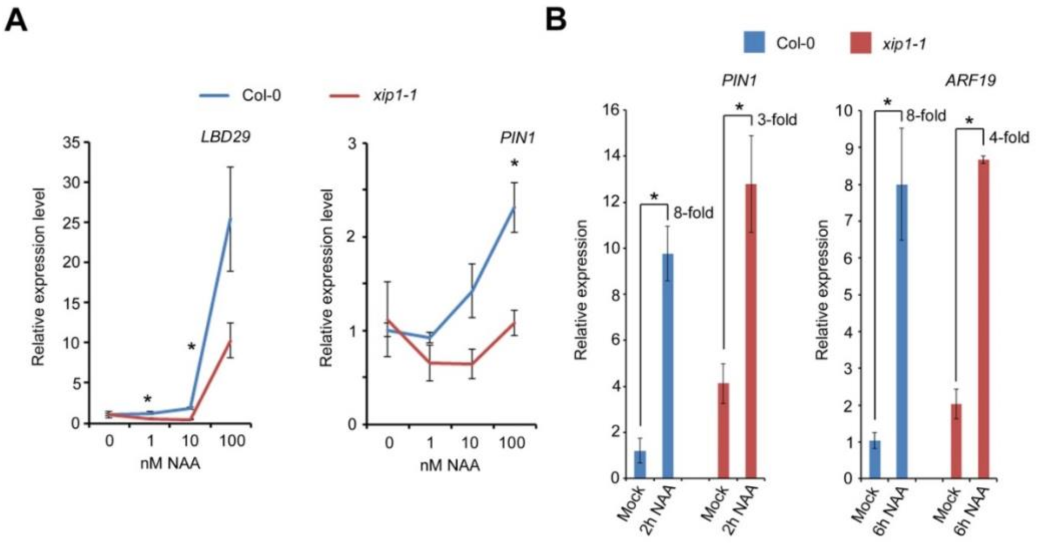

Figure S9. Auxin-inducible gene expression in *xip1-1*. **(A)** Auxin-inducible expression of *LBD29* and *PIN1* (as determined through qRT-PCR analysis) in Col-0 and *xip1-1* 5 day old seedling root tips treated with 0 (DMSO), 1, 10 or 100 nM NAA for 6 hours. Graphs show average ± standard error of 3 biological repeats. *, $p < 0.05$ according to Student's *t*-test compared to Col-0. **(B)** Auxin-inducible expression of *PIN1* and *ARF19* (as determined through qRT-PCR analysis) in Col-0 and *xip1-1* 7-day old seedling roots treated with mock (DMSO) or 1 μM NAA for indicated time (3 biological repeats). Graphs show average ± standard error (except d and f) of indicated sample numbers. *, $p \leqslant 0.05$ according to Student's *t*-test compared to mock. Fold change of mock versus NAA treatment is indicated in B.

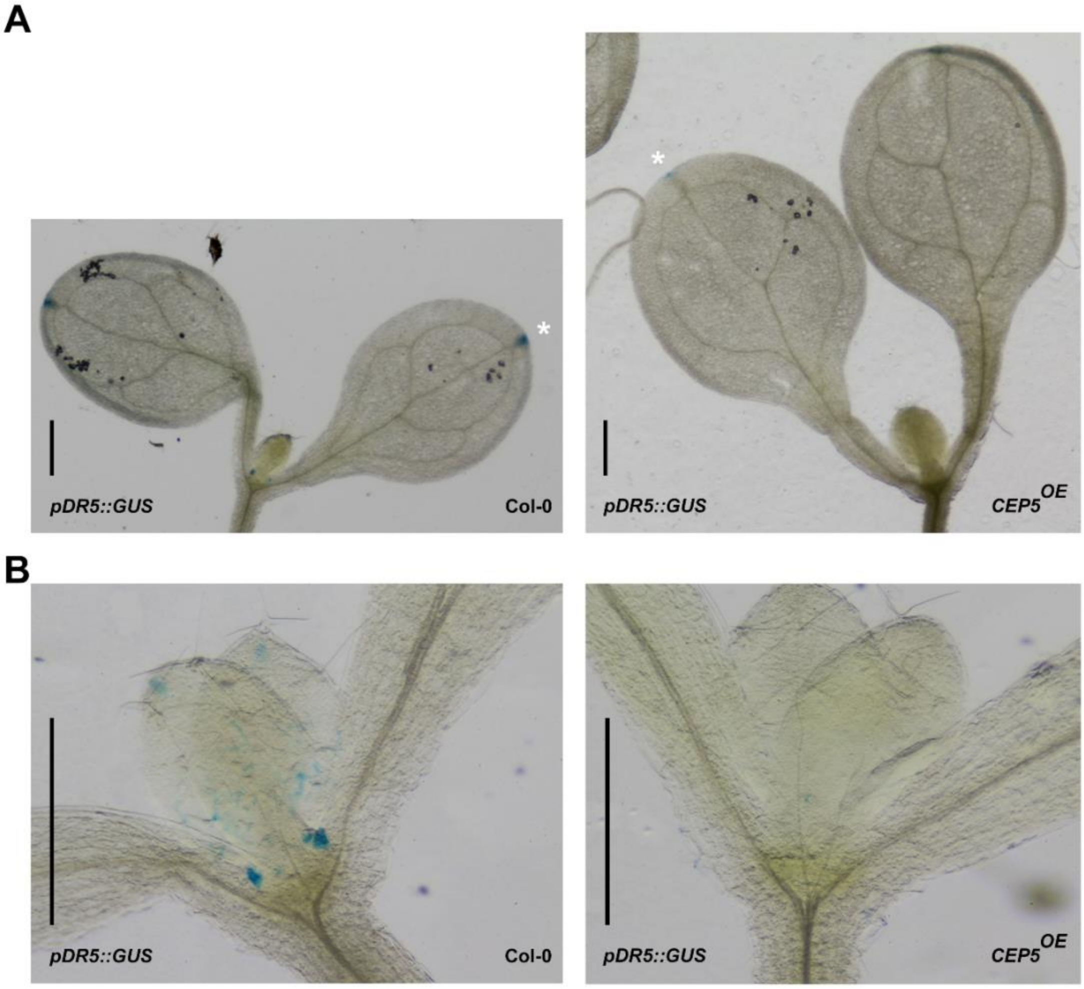

Figure S10. Representative pictures (selected from 26 seedlings) for *pDR5::GUS* activity in the cotyledons **(A)** and shoot apical meristem **(B)** of Col-0 and *CEP5_{OE}* at 3 days after germination**.** For (A), compare the staining at the position of the yellow asterisk. Scale bar, 0.5 mm.

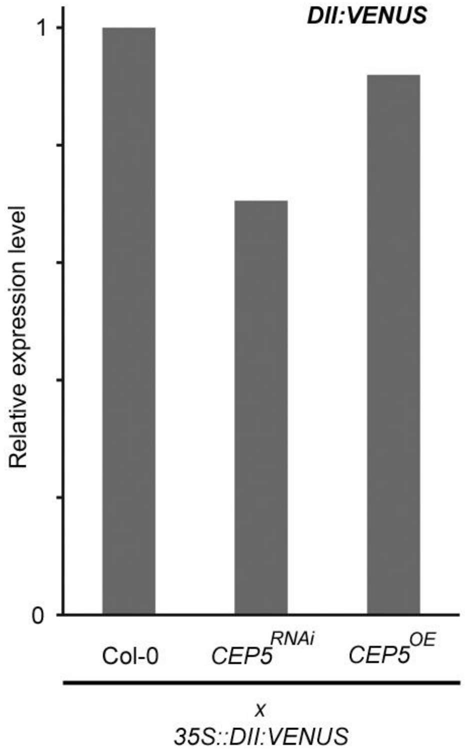

Figure S11. *DII:VENUS* transcriptional expression levels in F1 seedlings (pool of 5-10 seedlings at 6 days after germination) of Col-0 x *35S::DII:VENUS*, *CEP5$_{RNAi}$* x *35S::DII:VENUS*, and *CEP5$_{OE}$* x *35S::DII:VENUS* (graph compiled from 1 biological replicate).

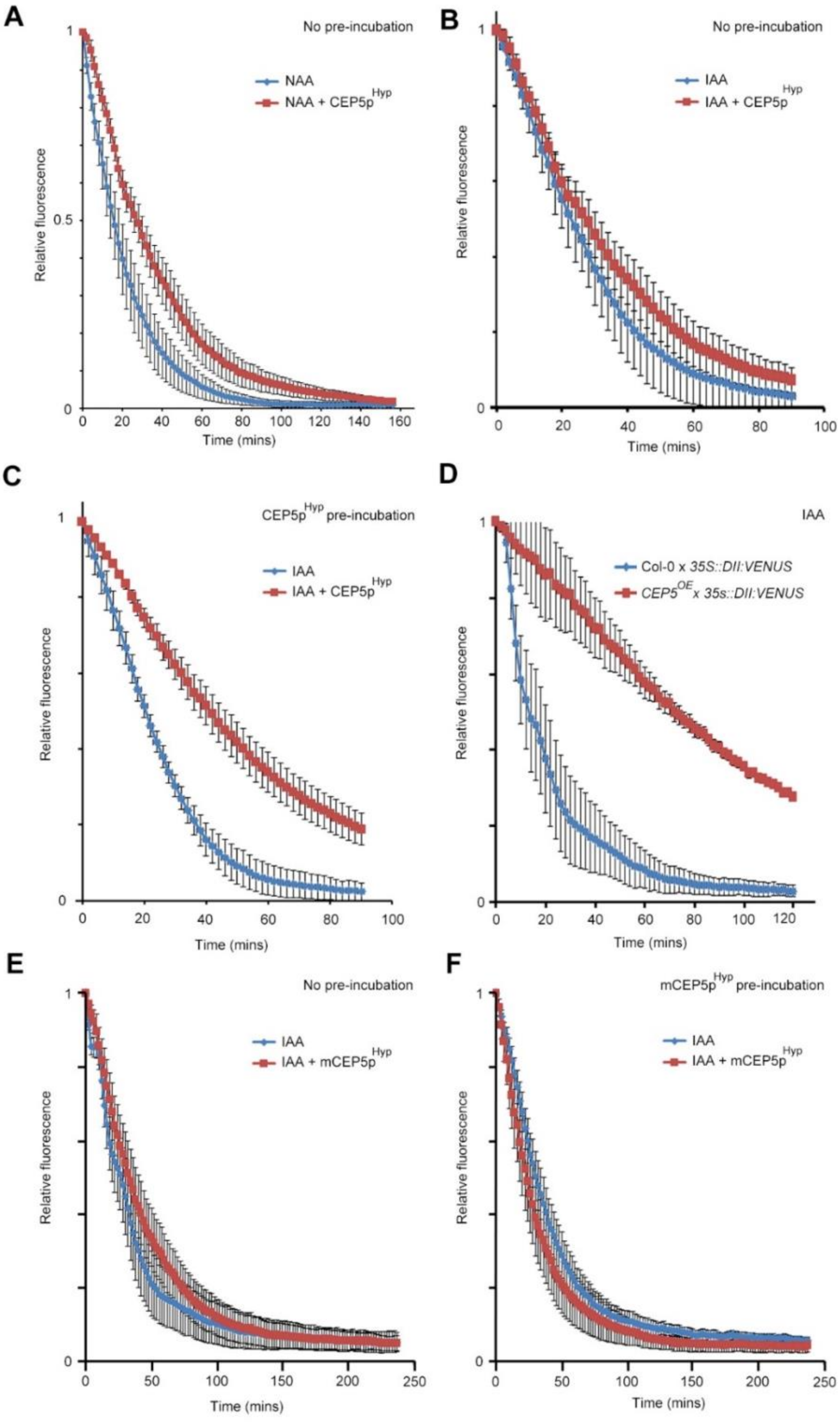

Figure S12. DII:VENUS fluorescence for indicated treatments (5 µM CEP5p$_{Hyp}$, 5 µM mCEP5p$_{Hyp}$, 1 µM IAA or 1 µM NAA) and in Col-0 (A,B,C,E,F) or *CEP5$_{OE}$* background (D). Pre-incubation was for 18 hrs. All graphs show average ± standard error of 2-5 biological replicates.

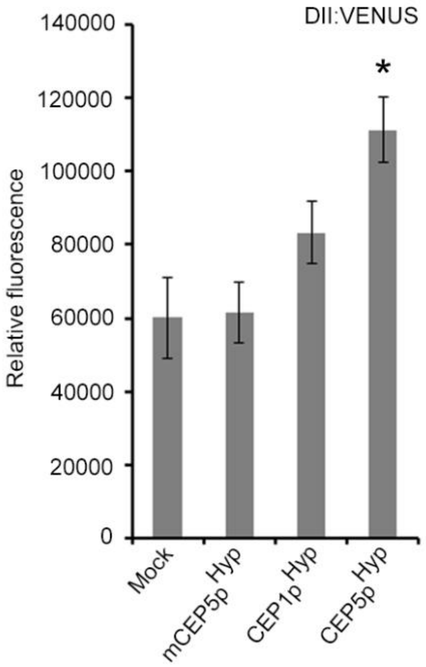

Figure S13. Relative DII:VENUS protein fluorescence in *35S::DII:VENUS* reporter line following 18 hrs incubation with 5 µM $CEP5p_{Hyp}$, $mCEP5p_{Hyp}$, or $CEP1p_{Hyp}$ (DFR[HYP]TNPGNS[HYP]GVGH) compared with mock (medium with water as used to dissolve CEPp) treatment at 5-6 days after germination (n = 11). Graph shows average ± standard error. *, $p < 0.05$ according to Student's *t*-test compared to mock and $mCEP5p_{Hyp}$.

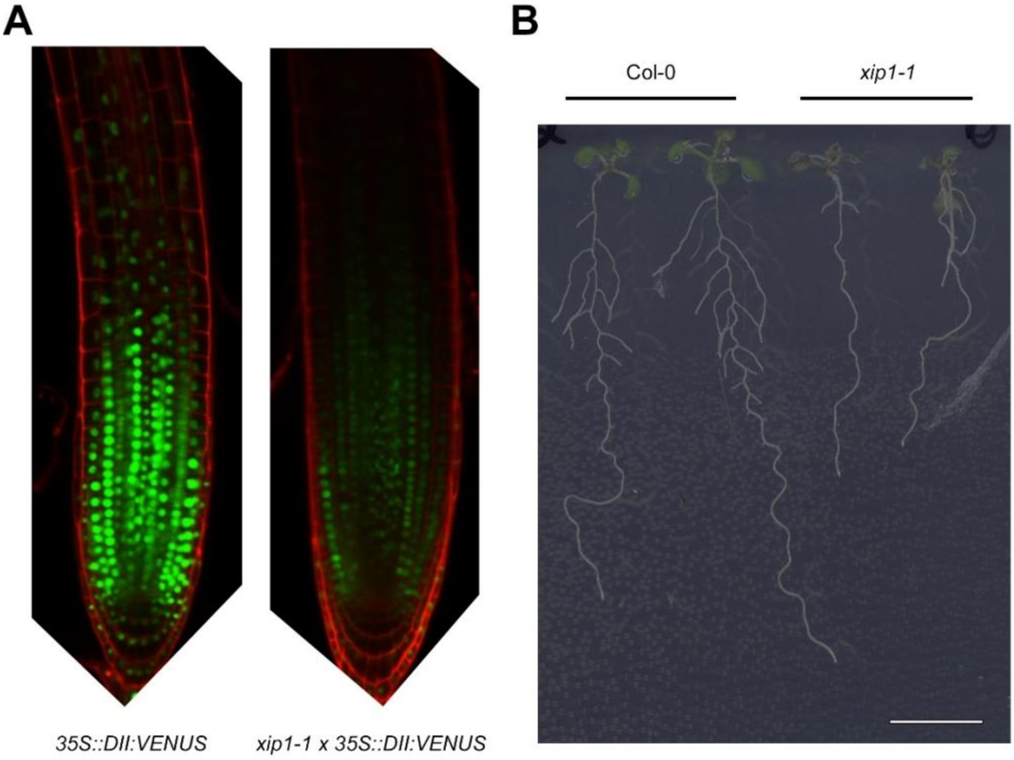

Figure S14. (A) Representative pictures of DII:VENUS in wildtype and *xip1*-1 background at 7 days after stratification (DAS). **(B)** Representative picture of Col-0 and *xip1-1* root architecture phenotype in 8-day-old seedlings. Scale bar, 1 cm.

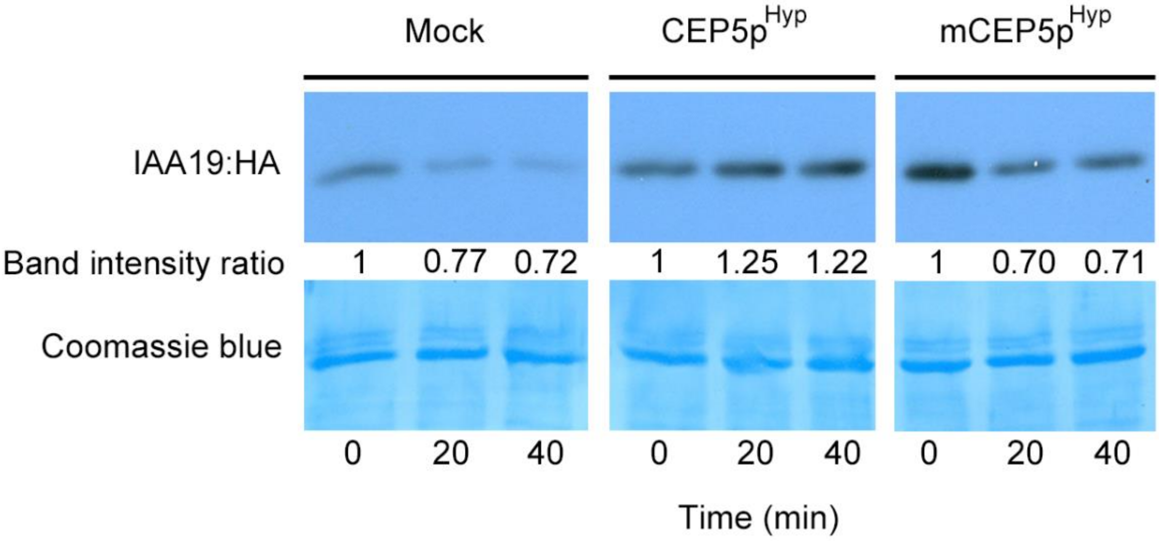

Figure S15. Representative Western blot of IAA19:HA levels in 10-day old seedlings grown in the presence of 5 µM CEP5p$_{Hyp}$ or 5 µM mCEP5p$_{Hyp}$ for indicated (short) times. The band intensity ratio, normalized to the loading control and relative to 0 min is indicated. Loading control is Coomassie blue. Mock refers to medium with water as used to dissolve (m)CEP5p$_{Hyp}$. *Note*: no auxin was used in this experiment.

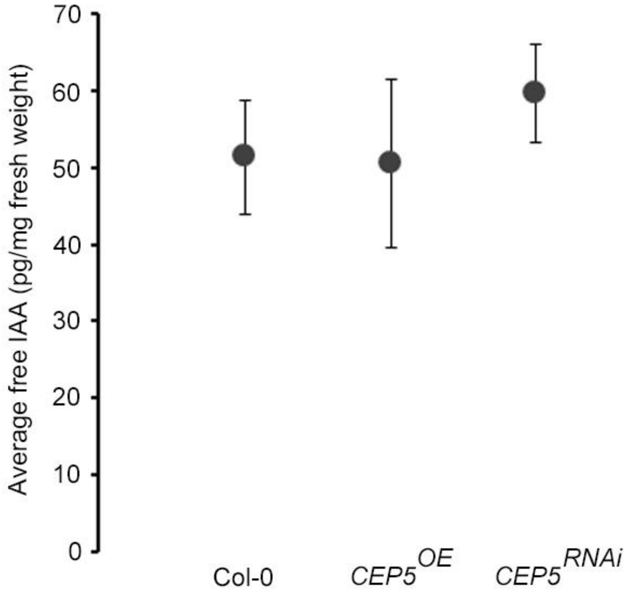

Figure S16. Free IAA levels in Col-0, *CEP5OE* and *CEP5RNAi* seedlings at 10 days after germination. Graph shows average ± standard error of 4-5 (mix of leaves and root material per sample) biological repeats. No significant differences were observed according to Student's *t*-test ($p > 0.4$).

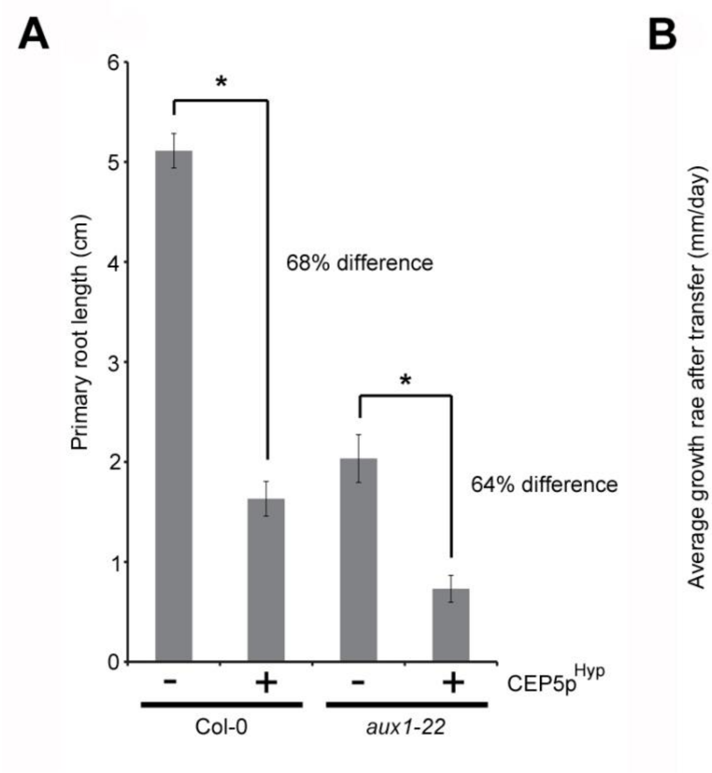

Figure S17. (A) Primary root length of Col-0 *Arabidopsis* seedlings under exogenous application of 5 μM CEP5p$_{Hyp}$ compared with mock treatment at 7 days after germination (n ⩾ 14 per condition). The % reduction in root length is indicated. *, $p < 0.05$ according to Student's *t*-test. **(B)** Primary root growth (mm/day) after transfer of 5-day old seedlings from ½ MS to ½ MS or 5 μM CEP5p$_{Hyp}$-containing medium for 3 days (n ⩾ 14 per condition). No significant differences were observed for CEP5p$_{Hyp}$-treated seedlings according to Student's *t*-test ($p < 0.05$). Graphs show average ± standard error.

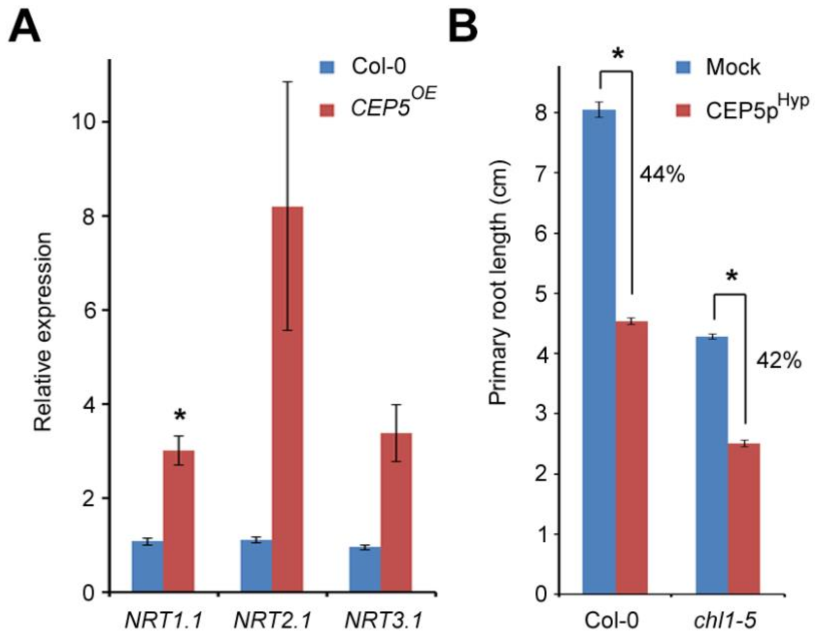

Figure S18. Evaluation of NRTs and CEP5. **(A)** *NRT* expression as determined by qPCR in Col-0 and $CEP5_{OE}$ seedling roots at 5 days after germination. *, $p < 0.05$ according to Student's *t*-test compared to Col-0. **(B)** Primary root length of Col-0 and *chl1-5* (an *NRT1* mutant allele) grown on medium containing mock (with water as used to dissolve CEPp) or 1 µM $CEP5p_{Hyp}$ at 10 days after germination ($n \geqslant 9$). *, $p < 0.05$ according to Student's *t*-test compared to mock. The % reduction in total lateral root density is indicated. All graphs show average ± standard error.

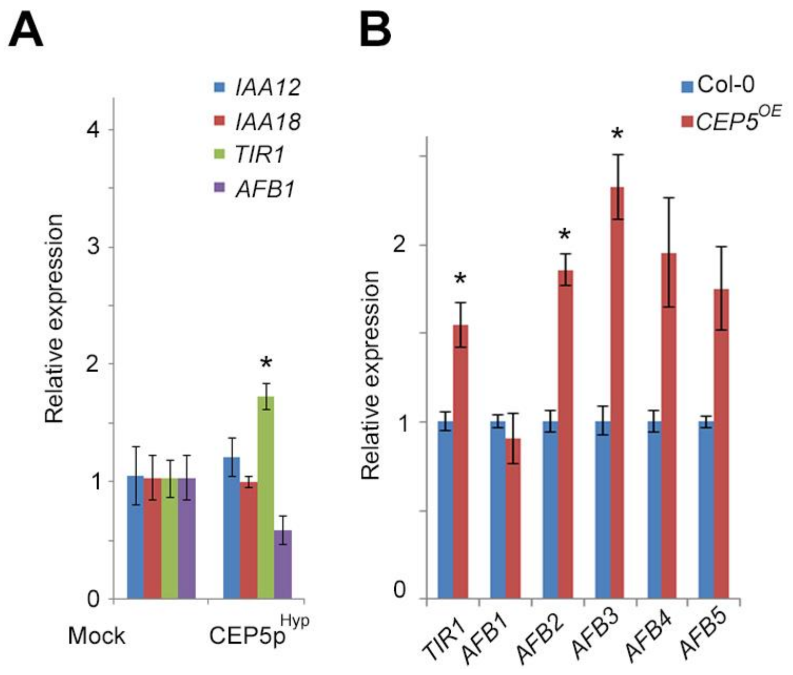

Figure S19. Expression levels of *TIR1/AFBs* and/or *AUX/IAAs* in 5 µM CEP5p$_{Pro}$ or CEP5p$_{Hyp}$-treated (overnight) 5-day old wild type seedlings (A) and in roots of *CEP5*$_{OE}$ 5-day old seedlings (B). *, p < 0.05 according to Student's t-test compared to mock (medium with water as used to dissolve CEP5p) (A) or Col-0 (B). All graphs show average ± standard error of 3 biological repeats.

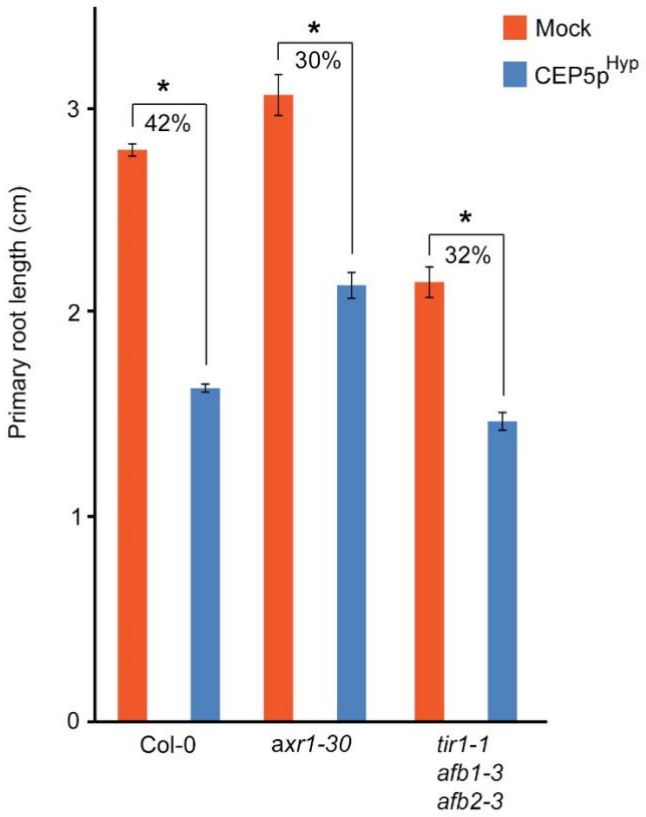

Figure S20. Primary root length in Col-0, *axr1-30* and *tir1-1 afb1-3 afb2-3* grown on ½ MS medium supplemented with mock (medium with water as used to dissolve CEP5p$_{Hyp}$) or 1 µM mCEP5p$_{Hyp}$. The relative reduction in primary root length is indicated. Graph shows average ± standard error. *, $p < 0.05$ according to Student's *t*-test compared to mock.

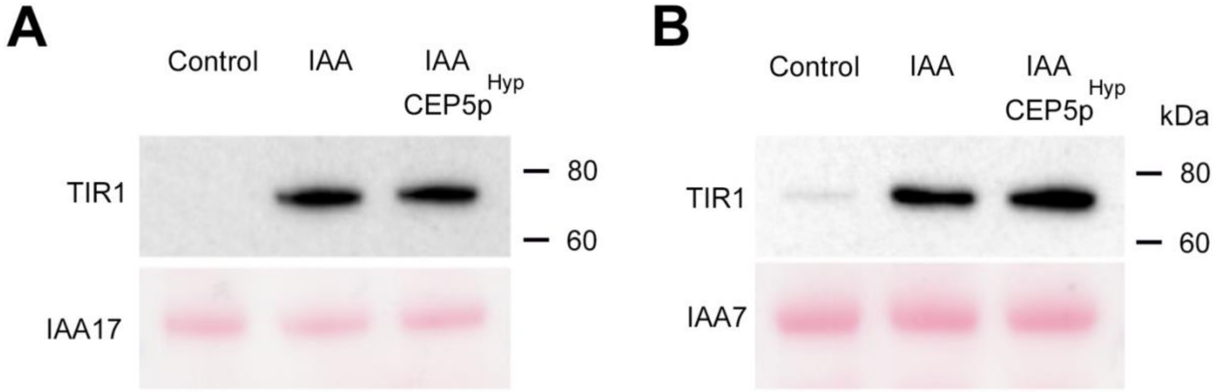

Figure S21. Western blot visualizing pull down of Biotin:IAA17 (A) or Biotin:IAA7 (B) with 3xFLAG:TIR1 in the presence of IAA or IAA + CEP5p$_{Hyp}$. IAA and CEP5p$_{Hyp}$ were used at concentrations of 1 µM and 10 µM, respectively.

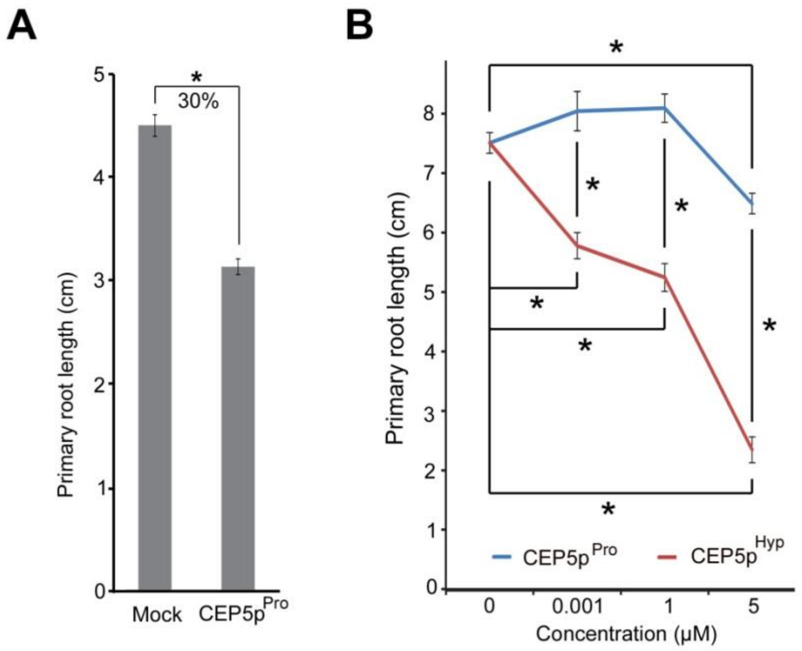

Figure S22. Bio-activity of CEP5p variants in primary root length assay. **(a)** Primary root length (cm) of Col-0 *Arabidopsis* seedlings under exogenous application of 1 μM $CEP5p_{Pro}$ compared with mock treatment at 7 days after germination (n ⩾ 164 per condition). The % reduction in root length is indicated. **(b)** Bioactivity of hydroxyprolinated CEP5 ($CEP5p_{Hyp}$: red line) in the primary root length assay for Col-0 compared with unmodified CEP5 ($CEP5p_{Pro}$: blue line) at 15 days after germination (n ⩾ 12 per condition). Graph shows average ± standard error. *, $p < 0.05$ according to Student's *t*-test as indicated.

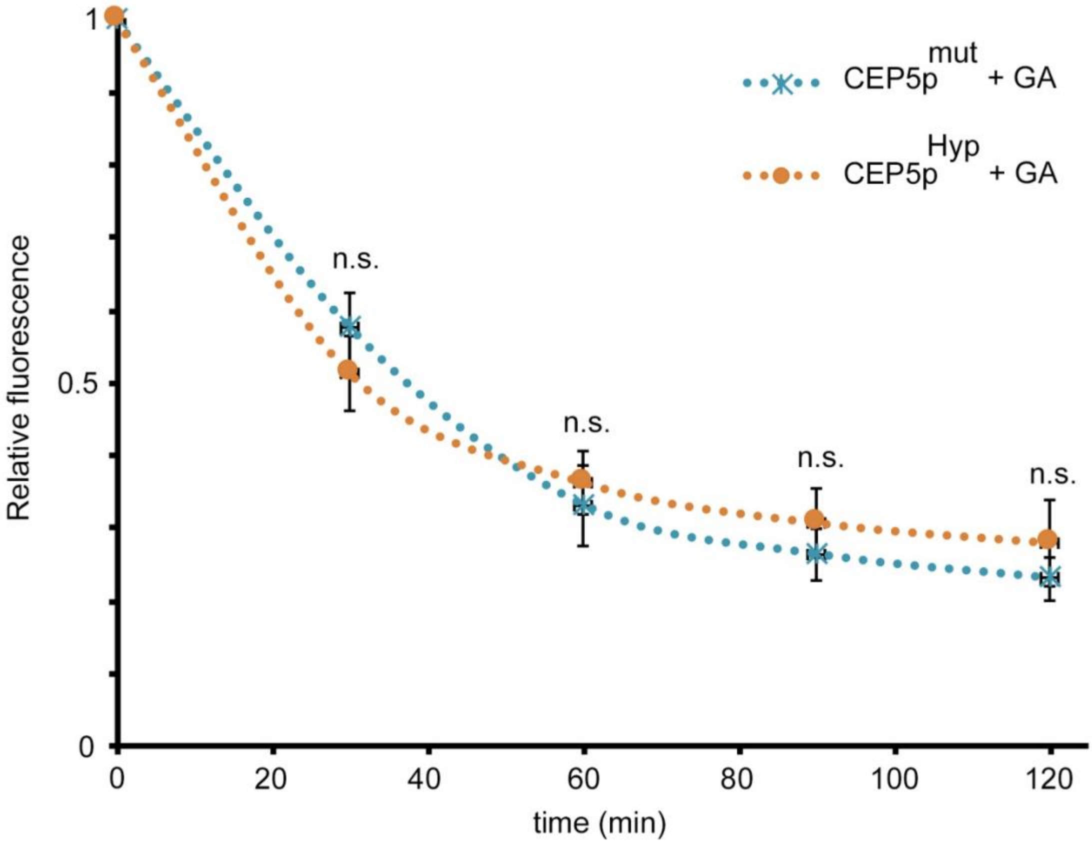

Figure S23. RGA:GFP fluorescence for indicated treatments (5 µM CEP5p$_{Hyp}$ + 1 µM GA or 5 µM mCEP5p$_{Hyp}$ + 1 µM GA) on an *pRGA::RGA:GFP* expressing line. Graphs shows average ± standard error of 6-7 biological replicates. n.s., Student's *t*-test compared within the time point did not reveal significant differences at $p < 0.05$.

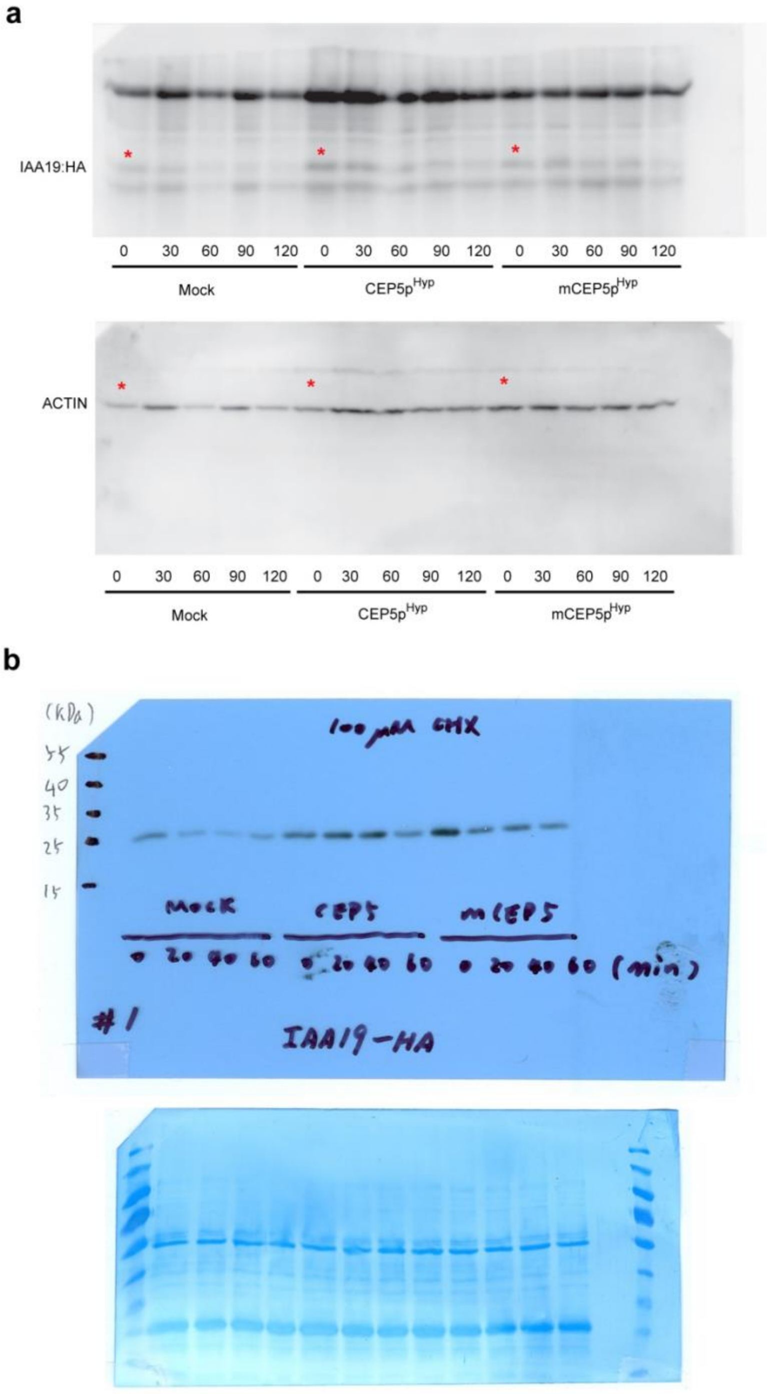

Figure S24. Original blots for Figure 4G (a) and Figure S15 (b). Lanes selected for Figure 3G are indicated with a red asterisk.

TABLE S1. Details and general information on most lines used in this study.

Arabidopsis **line**	**Type of line**	**Main Phenotype**	**References**
pCEP5::nGFP:GUS	reporter line	indicates that *CEP5* is expressed in both root and shoot tissues	[2]
CEP5$_{OE}$	overexpression line	*35S::CEP5* displays a shorter primary root and fewer lateral roots. Strong overexpression lines have smaller, often curled leaves	[2]
CEP5$_{RNAi}$	RNAi knockdown line	shows longer primary root, more stage I and II lateral root primordia, slight increase in plant height after post-bolting	[2]
35S::DII:VENUS	reporter line	protein-based reporter line for auxin levels. DII:VENUS provides a map of relative auxin distribution and is rapidly degraded in response to auxin	[3]
xip1-1	TLLING line	loss-of-function of CEP RECEPTOR 1. Displays shorter inflorescence stems, purple cotyledons and rosette leaves, defects in vascular organization, and reduced lateral root density	[4]
cepr2-3	loss-of-function line	T-DNA mutant of CEP RECEPTOR 2. Displays higher emerged lateral root density	[5]
pDR5:LUC	reporter line	reporter line for transcriptional auxin response	[6]
IAA19:HA	overexpression line	IAA19 is a AUX/IAA transcriptional repressor. This line expresses IAA19-HA fusion proteins which can be detected with anti-HA antibody	[7]
pBDL::BDL:GUS	reporter line	BDL is IAA12 from AUX/IAA family. This line expresses BDL-GUS fusion proteins	[8]
rpn12a-1	loss-of-function line	contains a mutation in a part of the 19S regulatory particle. Involved in complex assembly, decreased rates of leaf formation, reduced root elongation, delayed skotomorphogenesis	[9]
rpt2a-2	loss-of-function line	contains a mutation in a subunit of the 19S regulatory particle of the proteasome. Gates the axial channel of the 20S core particle and controls substrate entry and product release. It shows shorter roots, narrow serrated rosette leaves, stem fasciation and delayed flowing time	[9]
pRGA::GFP:RGA	complementing line	RGA, DELLA protein, repressor of GA signaling. This line rescues the phenotype caused by the *rga-24* null mutation, and GFP fluorescence was detected in the nuclei	[10]
chl1-5	loss-of-function line	NRT1.1 protein transports nitrate. The line is a knock-out mutant of NRT1.1 and shows resistance to the herbicide chlorate and a decrease in nitrate uptake	[11]
aux1-2	loss-of-function line	missense alleles, partical-loss-of-function of auxin influx transporter AUX1, shows partially agravitropic phenotype	[12]
aux1-22	loss-of-function line	splicing, null mutant of AUX1, shows agravitropic phenotype	[13]
pin2-2/eir1-1	loss-of-function line	a conserved G to A mutationat splice position -1 of auxin efflux transport PIN2, shows agravitropic phenotype	[14]
axr1-30	loss-of-function line	AXR1 encoding E1 enzyme which is essential for the activation SCF$_{TIR1/AFB}$ function. This line is a T-DNA mutation of AXR1 and shows auxin-resistant root growth	[15]
tir1-1 afb1-3 afb2-3	loss-of-function line	triple mutant of auxin receptors TIR1/AFBs and root elongation shows resistance to auxin treatment	[16]

SUPPLEMENTARY EXPERIMENTAL PROCEDURES

Plant growth and treatment conditions

Unless mentioned otherwise, seedlings were grown at 22°C under continuous light (110 µE m–2 s–1 photosynthetically active radiation, supplied by cool-white fluorescent tungsten tubes, Osram) on square Petri plates containing 50 ml solid half-strength MS growth medium supplemented with sucrose (per liter: 2.15 g MS salts, 0.1 g *myo*-inositol, 0.5 g MES, 8 g sucrose, 8 g plant tissue culture agar; pH adjusted to 5.7 with KOH). For peptide treatments, media was supplemented with CEP5p$_{Pro}$, CEP5p$_{Hyp}$, or mCEP5p$_{Hyp}$ peptide to a concentration as indicated in the text and/or figure legends. *35::DII:VENUS* seedlings were grown vertically on sugar-free ½ MS media (MS salts 2.15 g/L, Myo-inositol 0.1 g/L, MES 0.5 g/L, plant tissue culture agar 10 g/L, pH adjusted to 5.7) in 24 h light at 21°C. Growth conditions were as follows for data in 2i (12 DAG): *Arabidopsis thaliana* seedlings (Col-0) were grown vertically either on ½ MS agar (Sucrose 10 g/L, MS salts 2.15 g/L, Myo-inositol 0.1 g/L, MES 0.5 g/L, plant tissue culture agar 10 g/L, pH adjusted to 5.7) with or without 5 µM CEP5p$_{Hyp}$ or 5 µM mCEP5p$_{Hyp}$ in 24 h light at 21°C. For Col-0 x *35S:DII::VENUS*, *CEP5*$_{RNAi}$ x *35S:DII::VENUS*, *CEP5*$_{OE}$ x *35S:DII::VENUS*, the F1 generation was grown vertically on sugar-free ½ MS agar (MS salts 2.15 g/L, Myo-inositol 0.1 g/L, MES 0.5 g/L, plant tissue culture agar 10 g/L, pH adjusted to 5.7) until 5–6 DAG. For overnight DII:VENUS +/- IAA/NAA/CEP5 time-lapse experiments, DII:VENUS seedlings were grown vertically on sugar-free ½ MS media (see above) in 24 h light at 21°C until 5 DAG. At this time seedlings were transferred to ½ MS media supplemented with CEP5 5 µM or plain ½ MS media control plates and incubated overnight. For each biological replicate, the next day one seedling was selected for quantification and placed in a coverglass-bottom dish (Iwaki, Japan) and overlaid with squares of sugar-free ½ MS supplemented with either: IAA 1 µM, NAA 1 µM, IAA 1 µM + CEP5 5 µM, NAA 1 µM + CEP5 5 µM, or CEP5 5 µM alone. For non-time lapse comparison of fluorescence (DII:VENUS +/- CEP5), DII:VENUS seedlings were grown vertically on sugar-free ½

MS media (MS salts 2.15 g/L, Myo-inositol 0.1 g/L, MES 0.5 g/L, plant tissue culture agar 10 g/L, pH adjusted to 5.7) in 24 h light at 21°C until 5 DAG. At this time seedlings were transferred to ½ MS media supplemented with either CEP5 5 µM, or plain ½ MS media control plates and incubated overnight. The next day seedlings were mounted on glass slides. For analyses in **Figures 4A**, DII::VENUS seedlings were grown vertically either on sugar-free ½ MS agar (MS salts 2.15 g/L, Myo-inositol 0.1 g/L, MES 0.5 g/L, plant tissue culture agar 10 g/L, pH adjusted to 5.7), or sugar-free ½ MS agar supplemented with 5 µM CEP5p$_{Hyp}$ in 24 h light at 21°C until 5–6 DAG. For analyses in **Figures 4D-E**, DII::VENUS seedlings were grown vertically on sugar-free ½ MS agar (MS salts 2.15 g/L, Myo-inositol 0.1 g/L, MES 0.5 g/L, plant tissue culture agar 10 g/L, pH adjusted to 5.7), in 24 h light at 21°C until 5–6 DAG. Seedlings were then treated with sugar-free ½ MS agar supplemented with either NAA 1 µM or NAA 1 µM + CEP5p$_{Hyp}$ 5 µM whilst being imaged on a Leica SP5 confocal microscope (Leica, Wetzlar, Germany (514 nm detector: gain value 100%, offset value 28.98). For short term CEP5p treatments, DII:VENUS seedlings were grown vertically on sugar-free ½ MS media (MS salts 2.15 g/L, Myo inositol 0.1 g/L, MES 0.5 g/L, plant tissue culture agar 10 g/L, pH adjusted to 5.7) in 24 h light at 21°C until 5 DAG in square petri dishes. After this time seedlings were placed in coverglass-bottom dishes (Iwaki, Japan) and overlaid with squares of sugar-free ½ MS containing either 5 µM CEP5p$_{Hyp}$ or mCEP5$_{Hyp}$, or unsupplemented fresh media as a control. For determination of IAA19 protein stability, homozygous plants expressing *35S::IAA19:HA* were grown in 1/2 B5 media containing DMSO, CHX, 5 µM CEP5p$_{Hyp}$ or mCEP5p$_{Hyp}$ for 10 days or the indicated minutes. Synthetic peptides were obtained from GenScript (www.genscript.com/peptide-services.html?src=home). For auxin inducibility analyses in **Figures 3E and S9**, seedlings were grown on nylon mesh and transferred to auxin-containing medium for 2 or 6 hours. For the following experiments, seedlings were grown on half-strength MS medium containing 1% sucrose at 21℃ under 110 µmol m^{-2}s^{-1} light. Rosette area analysis under osmotic stress treatment was performed as described previously [17]. Wild type and mutant seeds were equally distributed on a 14

cm-diameter petri dish and seedlings were grown on half-strength MS with or without 50 mM D-mannitol (plant culture tested; Sigma) under a 16-h-day and 8-h-night regime. For expression analysis under mannitol stress, seedlings were grown on square petri plates under continuous light. For treatments involving transfer, the growth medium was overlaid with nylon mesh (Prosep) of 20 µm pore size to facilitate transfer. For in-soil plant growth-survival assay was performed as described previously with slight modifications [18]. Wild type and mutant plants were randomized in the same tray for 18-21 days before the weight of all pots was equalized. Water was withheld for approximately 2 weeks and then re-watered. Plants (17 to 23) of each genotype were used to assess survival in three independent experiments. For phosphoproteome analyses, seedlings were grown on a rotational shaker (90 rpm) in liquid culture [half-strength Murashige and Skoog (MS)] for 5 days after germination at 21°C under a 16-h-day and 8-h-night regime.

qPCR analyses

RNA was extracted by first performing an RNA extraction with TRI Reagent® from Sigma-Aldrich according to the manufacturer's protocol, followed by a DNAse treatment on the isolated RNA (DNase I recombinant RNase-free from Roche Applied Science). This was followed by an extra RNA extraction procedure with the Plant RNeasy Mini kit from Qiagen according to the manufacturer's protocol to further clean up the RNA. Next, 1 µg of total RNA was used for cDNA synthesis using the iScript kit from Bio-Rad according to the manufacturer's protocol. The real-time qRT-PCR reaction was carried out on the LightCycler 480 from Roche Applied Science with the LightCycler 480 SYBR Green I Master Mix from Roche Applied Science. For analysis of stress-related genes expression, normalization was done against housekeeping genes *ACTIN* and *EEF*. Primers used in this study are as follows: *ACTIN* (GGCTCCTCTTAACCCAAAGGC and CACACCATCACCAGAATCCAGC), *EEF* (CTGGAGGTTTTGAGGCTGGTAT and CCAAGGGTGAAAGCAAGAAGA),

CEP5 (CCATGGACGAACCCTAAAAG and TGCCATCATCGTCTTGCTAT), *ERF5* (AAATTCGCGGCGGAGATTCGTG and TCAAACGTCCCAAGCCAAACGC), *ERF6* (TCGAATCCTCCTCGCGTTACTG and TTCGGTGGTGCGATCTTCAACG, *STZ* (TCACAAGGCAAGCCACCGTAAG and TTGTCGCCGACGAGGTTGAATG). For the analysis of *CEP5* expression after auxin treatment, the expression of *CEP5* (CCATGGACGAACCCTAAAAG and TGCCATCATCGTCTTGCTAT), *TIR1* (GCCTCTCTCTATCTGGCCTCTTGAC and AGGGCAGCTCTCTGGTCTCGAGTCC), *AFB1* (AGTGATTTGATGCTTCATCACTTGT and CAATGACTTCGACATTGAGCCTTGGCA), *AFB2* (TGGCGGCGCATCCATTCTTGTCCCA and AGATGCTCTCCATAGCCTTTGCAGG), *AFB3* (AGCTCGAGATGCTTTCGATAGCTTTTG and TCATTCTGTTCCATCCCATTATTCTCA), *AFB4* (TCTCATATCCCGTGGAGGCT and CATGCAGTGTACCTGAAGCA), *AFB5* (GCTGCAAGGATATTGCACGAG and GCATTCCTCCCAAGTCCCAA), *IAA12* (GGTACTACTTGTCGAGAAAGGTTAAACC and CCCCTTCCTTATCTTCATAAGTGAGTAC), *IAA18* (TGATGATTCCACAAGAGAGAAG and CACCAGGTGGTCCAAGC), *LBD16* (AGACGTCAGCCGCCGGAGAT and GCGAGCTCTGTGGCGAGACC), *LBD18* (GGCCACAGCCTATGCCGCAA and AGCCCCACGGCGACAGAAGA), *LBD29* (GCTAGGCTTCAAGATCCCATC and TGTGCTGCTTGTTGCTTTAGA), *ARF19* (TCCAAGTTCCAACGAAGGAG and AAACTAAAGGCCCTGCACAA), *PIN1* (TACTCCGAGACCTTCCAACTACG and TCCACCGCCACCACTTCC), *NRT1.1* (GCACATTGGCATTAGGCTTT and CTCAATCCCCACCTCAGCTA), *NRT2.1* (AACAAGGGCTAACGTGGATG and CTGCTTCTCCTGCTCATTCC) and *NRT3.1* (GGCCATGAAGTTGCCTATG and TCTTGGCCTTCCTCTTCTCA) was determined using two or three biological repeats and the reference genes *EEF-1α4* (CTGGAGGTTTTGAGGCTGGTAT and CCAAGGGTGAAAGCAAGAAGA) and

At2g32170 (GGACCTCTGTTGTATCATTTTGCG and CAACCCTCTTTACATCCTCCAAAC). To assess *DII:VENUS* levels in *CEP5*$_{OE}$, RNA was extracted from a pool of at least 5-10 F1 seedlings at 6 DAG s. cDNA was subsequently prepared from a minimum of 250 ng RNA (determined by UV spectrophotometry) using a SuperScript II reverse transcriptase kit and Oligo(dT)12–18 primers (Invitrogen, USA), according to the manufacturer's instructions. Primers for the N7 nuclear localisation sequence of *VENUS* (GGACTCTGAGGATGGAAACG and TCAGCTTCTGTGTCGTCAATG) and referenced to *ACTIN* (CTGGA GGTTTTGAGGCTGGTAT and CCAAGGGTGAAAGCAA GAAGA). Eight technical repeats (done as 2 sets of 4 but all from the same pooled sample) were performed.

References

1. Ljung K (2013) Auxin metabolism and homeostasis during plant development. Development 140:943-50. doi: 10.1242/dev.086363 (https://dev.biologists.org/content/140/5/943)

2. Roberts I, Smith S, De Rybel B, Van Den Broeke J, Smet W, De Cokere S, Mispelaere M, De Smet I and Beeckman T (2013) The CEP family in land plants: evolutionary analyses, expression studies, and role in Arabidopsis shoot development. J Exp Bot 64:5371-81. doi: 10.1093/jxb/ert331

3. Brunoud G, Wells DM, Oliva M, Larrieu A, Mirabet V, Burrow AH, Beeckman T, Kepinski S, Traas J, Bennett MJ and Vernoux T (2012) A novel sensor to map auxin response and distribution at high spatio-temporal resolution. Nature 482:103-6. doi: 10.1038/nature10791

4. Bryan AC, Obaidi A, Wierzba M and Tax FE (2012) XYLEM INTERMIXED WITH PHLOEM1, a leucine-rich repeat receptor-like kinase required for stem growth and vascular development in Arabidopsis thaliana. Planta 235:111-22. doi: 10.1007/s00425-011-1489-6

5. Dimitrov I and Tax FE (2018) Lateral root growth in Arabidopsis is controlled by short and long distance signaling through the LRR RLKs XIP1/CEPR1 and CEPR2. Plant Signal Behav 13:e1489667. doi: 10.1080/15592324.2018.1489667

6. Moreno-Risueno MA, Van Norman JM, Moreno A, Zhang J, Ahnert SE and Benfey PN (2010) Oscillating gene expression determines competence for periodic Arabidopsis root branching. Science 329:1306-11. doi: 10.1126/science.1191937

7. Cho H, Ryu H, Rho S, Hill K, Smith S, Audenaert D, Park J, Han S, Beeckman T, Bennett MJ, Hwang D, De Smet I and Hwang I (2014) A secreted peptide acts on BIN2-mediated phosphorylation of ARFs to potentiate auxin response during lateral root development. Nat Cell Biol 16:66-76. doi: 10.1038/ncb2893

8. Weijers D, Schlereth A, Ehrismann JS, Schwank G, Kientz M and Jurgens G (2006) Auxin triggers transient local signaling for cell specification in Arabidopsis embryogenesis. Dev Cell 10:265-70. doi: 10.1016/j.devcel.2005.12.001

9. Ustun S, Sheikh A, Gimenez-Ibanez S, Jones A, Ntoukakis V and Bornke F (2016) The Proteasome Acts as a Hub for Plant Immunity and Is Targeted by Pseudomonas Type III Effectors. Plant Physiol 172:1941-1958. doi: 10.1104/pp.16.00808

10. Silverstone AL, Jung H-S, Dill A, Kawaide H, Kamiya Y and Sun T-p (2001) Repressing a Repressor:Gibberellin-Induced Rapid Reduction of the RGA Protein in

Arabidopsis. The Plant Cell 13:1555. doi: 10.1105/TPC.010047

11. Yi-Fang Tsay, Julian I. Schroeder, Kenneth A. Feldmann and Crawford NM (1993) The Herbicide Sensitivity Gene CM.1 of Arabidopsis Encodes a Nitrate-Inducible Nitrate Transporter. Cell 72:705-713.

12. Swarup R, Kargul J, Marchant A, Zadik D, Rahman A, Mills R, Yemm A, May S, Williams L, Millner P, Tsurumi S, Moore I, Napier R, Kerr ID and Bennett MJ (2004) Structure-function analysis of the presumptive Arabidopsis auxin permease AUX1. Plant Cell 16:3069-83. doi: 10.1105/tpc.104.024737

13. Bennett MJ, Marchant A, Green HG, May ST, Ward SP, Millner PA, Walker AR, Schulz B and Feldmann KA (1996) Arabidopsis AUX1 gene: a permease-like regulator of root gravitropism. Science 273:948-50. doi: 10.1126/science.273.5277.948

14. Luschnig C, Gaxiola RA, Grisafi P and Fink GR (1998) EIR1, a root-specific protein involved in auxin transport, is required for gravitropism in Arabidopsis thaliana. Genes Dev 12:2175-87. doi: 10.1101/gad.12.14.2175

15. Gilkerson J, Hu J, Brown J, Jones A, Sun TP and Callis J (2009) Isolation and characterization of cul1-7, a recessive allele of CULLIN1 that disrupts SCF function at the C terminus of CUL1 in Arabidopsis thaliana. Genetics 181:945-63. doi: 10.1534/genetics.108.097675

16. Parry G, Calderon-Villalobos LI, Prigge M, Peret B, Dharmasiri S, Itoh H, Lechner E, Gray WM, Bennett M and Estelle M (2009) Complex regulation of the TIR1/AFB family of auxin receptors. Proc Natl Acad Sci U S A 106:22540-5. doi: 10.1073/pnas.0911967106

17. Claeys H, Van Landeghem S, Dubois M, Maleux K and Inze D (2014) What Is Stress? Dose-Response Effects in Commonly Used in Vitro Stress Assays. Plant Physiol 165:519-527. doi: 10.1104/pp.113.234641

18. Skirycz A, Vandenbroucke K, Clauw P, Maleux K, De Meyer B, Dhondt S, Pucci A, Gonzalez N, Hoeberichts F, Tognetti VB, Galbiati M, Tonelli C, Van Breusegem F, Vuylsteke M and Inze D (2011) Survival and growth of Arabidopsis plants given limited water are not equal. Nat Biotechnol 29:212-4. doi: 10.1038/nbt.1800
